# Dissecting the Characteristics and Dynamics of Human Protein Complexes at Transcriptome Cascade Using RNA-Seq Data

**DOI:** 10.1371/journal.pone.0066521

**Published:** 2013-06-18

**Authors:** Geng Chen, Jiwei Chen, Caiping Shi, Leming Shi, Weida Tong, Tieliu Shi

**Affiliations:** 1 The Center for Bioinformatics and Computational Biology, Shanghai Key Laboratory of Regulatory Biology, the Institute of Biomedical Sciences and School of Life Sciences, East China Normal University, Shanghai, China; 2 National Center for Toxicological Research, US Food and Drug Administration, Jefferson, Arkansas, United States of America; University of Saarland Medical School, Germany

## Abstract

Human protein complexes play crucial roles in various biological processes as the functional module. However, the expression features of human protein complexes at the transcriptome cascade are poorly understood. Here, we used the RNA-Seq data from 16 disparate tissues and four types of human cancers to explore the characteristics and dynamics of human protein complexes. We observed that many individual components of human protein complexes can be generated by multiple distinct transcripts. Similar with yeast, the human protein complex constituents are inclined to co-express in diverse tissues. The dominant isoform of the genes involved in protein complexes tend to encode the complex constituents in each tissue. Our results indicate that the protein complex dynamics not only correlate with the presence or absence of complexes, but may also be related to the major isoform switching for complex subunits. Between any two cancers of breast, colon, lung and prostate, we found that only a few of the differentially expressed transcripts associated with complexes were identical, but 5–10 times more protein complexes involved in differentially expressed transcripts were common. Collectively, our study reveals novel properties and dynamics of human protein complexes at the transcriptome cascade in diverse normal tissues and different cancers.

## Introduction

Proteins usually exert their functions through cooperative interaction with other proteins and the protein-protein interactions are crucial for myriad biological processes [1]. Protein interaction networks have topological and dynamic properties, and the interruptions of the networks may lead to certain diseases [2]–[4]. The protein complexes are basic representatives of the interaction networks, which allow a number of proteins to join together to execute various vital cellular tasks [5]. In yeast, a significantly large fraction of protein-coding genes are involved in the formation of protein complexes [6], [7]. Over one thousand human protein complexes have also been experimentally validated so far [8]. Several previous researches suggested that human diseases are closely associated with protein complexes [9], [10]. However, little is known about the expression features of human protein complexes in different human normal and disease tissues at the transcriptome cascade.

Proteins are translated from their corresponding transcripts which are produced by associated genes. A number of prior studies advocated that gene/mRNA expression level could correlate well with protein abundance based on the estimation from RNA-Seq and mass spectrometry (MS) data [11]–[14]. Furthermore, these researches suggested that the reason for lower correlations between the gene/mRNA and protein levels observed in other studies [15]–[19] was likely due to the platform limitations and/or defects for expression quantification methods. Certainly, the mRNA stability and translation efficiency can also influence the protein abundance [13]. Owing to the protein abundance is influenced by multiple factors, it is still unclear the exact correlation between the expression levels of transcripts and their corresponding protein products. If all the associated transcripts of the protein complex subunits expressed, those constituent proteins could be encoded as well and under certain condition they will be assembled into corresponding complexes to exert specific functions. Otherwise, if any one of the constituents of certain protein complex was absent, this protein complex would not be formed properly with dysfunction. Due to proteins are encoded by transcripts, the expression profile of the transcripts for corresponding protein complex components represent important properties of complexes. Since RNA-Seq enables the global view of gene and transcript activities with great sensitivity and specificity [20]–[25], the transcriptome sequencing data promise great opportunities to explore the associated transcript expression profile of protein complex components.

Nevertheless, few studies have investigated the dynamic expression profiles of human protein complexes at the transcriptome cascade. Although Bossi and Lehner have inquired the human protein interactions using the microarray expression data [26], they conducted the study at the gene level rather than at the isoform level. Owing to gene could yield a number of transcript variants (protein-coding and/or non-protein-coding) through alternative splicing [27], [28] and/or alternative transcription (alternative transcriptional initiation and/or termination) [29], a single gene usually encode multiple different proteins under various tempo-spatial conditions [30]. Here, we interrogated the expression properties and dynamics of human protein complexes in diverse human tissues and human cancers using corresponding RNA-Seq data. We found that a protein could be encoded by several distinct transcripts and protein complexes shared constituents are common for human. Most of the transcripts that associated with protein complex components were found expressed in 16 different human tissues, moreover, the major isoform of the genes from protein complexes are inclined to involve in the complex formation. We also observed that although several differentially expressed transcripts of protein complex components are the same between any two cancers of breast, colon, lung and prostate, much more common protein complexes that related to differentially expressed transcripts were found. Our results reveal novel and important characteristics and dynamics of human protein complexes in diverse normal tissues and different cancers at the transcriptome cascade.

## Materials and Methods

### Mapping Human Protein Complex to Ensembl Transcriptome

We downloaded the experimentally validated human protein complexes from the CORUM (the Comprehensive Resource of Mammalian protein complexes) database [8], http://mips.helmholtz-muenchen.de/genre/proj/corum/. The redundant protein complexes were removed and complexes that contain uncertain subunits (127 complexes) were also excluded. We downloaded the human transcriptome set from Ensembl database [31] (Ensembl 69, equivalent to GENCODE [32] version 14) and the ID mapping information for Uniprot proteins to Ensembl transcriptome from Uniprot database (http://www.uniprot.org/). We removed the alternative haplotype/supercontig entries from the Ensembl human transcriptome. Then we mapped the human protein complex components to the Ensembl genes and transcripts based on the Uniprot ID mapping annotation. The protein complexes that all of their constituents could be found associated Ensembl genes and transcripts were kept and those ones that do not meet the criteria were eliminated (Supplementary Table S1).

### RNA-Seq Data Set Used

We used the high throughput transcriptome sequencing data of 16 different human tissues from Illumina Human Body Map (HBM) 2.0 project (www.illumina.com; ArrayExpress ID: E-MTAB-513) to explore the expression profile of human genes and transcripts. We also used the transcriptome sequencing data from four human cancers of breast [33], colon, lung [34] and prostate [35], and their related control samples as well. The corresponding information of these RNA-Seq data could be found in Supplementary Table S2. These datasets were then used in the subsequent analyses for human protein complexes.

### Gene and Transcript Expression Quantification

All the RNA-Seq datasets we used were separately aligned onto the Ensembl human transcriptome using Bowtie (version 0.12.8) [36]. In order to enable each mapped read to find its optimal alignments and take the multi-mapping hits into account as well, we employed the parameters of “-a –best –strata -S -m 100 -X 500–chunkmbs 256” for Bowtie. By default, the Bowtie aligner allows two mismatches in the seed matching. We next followed the MMSEQ [37] (version 0.11.2) pipeline to quantify the expression of Ensembl genes and isoforms using the RNA-Seq data of each sample step by step. More details about MMSEQ pipeline could be found on the website: http://bgx.org.uk/software/mmseq.html.

### Differential Expression Calling

To carry out differential expression for the four human cancers of breast, colon, lung and prostate, we first used the R script (readmmseq.R) in the MMSEQ package to obtain the normalized (by their median deviation from the mean) aligned read counts for each Ensembl transcript. Then, we conducted the differential expression analysis of isoforms between cases and controls for four cancers using NOISeq [38]. According to the number of experimental replicates, read length and the sequencing depth for each cancer (Supplementary Table S1), we used distinct thresholds that are appropriate for these four human cancers to determine the differentially expressed transcripts. The biological replicate number is very important for the differential expression calling. Therefore, if the tested cancer data set has less replicates, a more stringent criterion will be used in differential expression analysis. There is no replicate for the lung cancer data set, while the breast and colon cancer data sets separately contain 2 biological replicates. There are 9 controls and 12 cases for the prostate cancer data set. To make the differential expression results more reliable, we used different thresholds of “q” for the cancers of breast, colon, lung and prostate, which are 0.9, 0.9, 0.99 and 0.8, respectively.

## Results

### Mapping Human Protein Complexes to Human Genes and Transcripts

**Figure 1:**
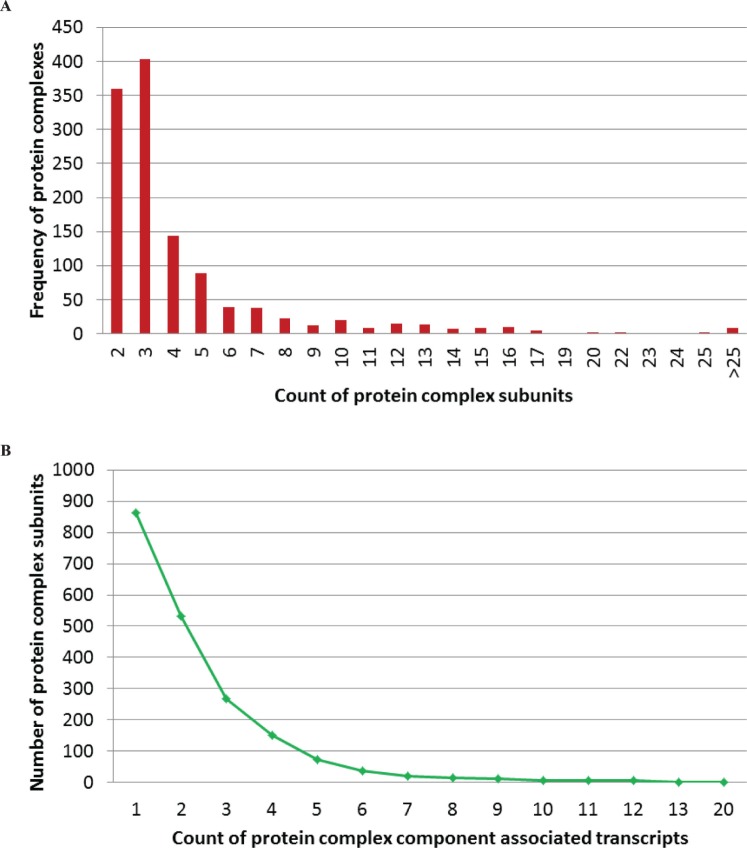
Characteristics of human protein complexes. (A) Distribution of the number of components for human protein complexes. (B) Distribution of the count of associated transcripts for human protein complex subunits.

To carry out this study, we first mapped the experimentally validated human protein complexes obtained from CORUM database [8] to the Ensembl human genes and transcripts [31]. The components of 1213 non-redundant protein complexes were unambiguously mapped to Ensembl human transcriptome (Ensembl 69, corresponding to GENCODE [32] version 14) according to the Uniprot (Universal Protein Resource) ID mapping annotation (Supplementary Table S1). These protein complexes are made up of 1986 proteins and involved in 2012 Ensembl genes and 4488 Ensembl transcripts, and they mainly comprise 2–4 protein constituents (Fig. 1A and B). More than half (56.6%) of the protein complex subunits individually possess at least 2 associated transcripts and even > = 2 corresponding genes, moreover, these components are associated with 93.32% of the 1213 human protein complexes. On average, each complex constituent has 2.26 and 1.01 associated transcripts and genes, respectively. In most of the cases, several distinct isoforms from the same gene generate the identical protein and these transcripts are produced through alternative transcription and/or alternative splicing (common coding sequence (CDS) regions but different untranslated regions (UTRs)). Only in a few of the cases, one protein could be encoded by different genes. However, by contrast, only about 17.5% of the Uniprot proteins that could be mapped to Ensembl transcriptome possess > = 2 Ensembl transcripts and 1.35 transcripts per protein on average, which are both significantly lower than that of protein complex components. The results show that one human protein may be encoded by distinct transcripts/genes, and the protein complex constituents are a fraction of proteins that prone to individually hold at least 2 related transcripts. We also observed that a notable fraction of protein complexes only differ in one or two subunits. Among those 1986 proteins from complexes, about half (49.09%) of them are shared across at least two different complexes, protein P05556 is the highest, which is the component of 48 protein complexes. Of the 1213 complexes, no case is distinct proteins generated by the same gene are involved in disparate complexes. All the complexes are comprised of proteins from different genes, except the molybdopterin synthase complex [39] is made of two distinct proteins encoded by the same gene.

### Expression Quantification of Genes and Isoforms Using RNA-Seq Data

To study the expression profiles of human protein complexes at the transcriptome cascade, we collected the RNA-Seq data from 16 different human normal tissues (Illumina Human Body Map 2.0 project) and 4 groups of cases and controls for the human cancers of breast [33], colon, lung [34] and prostate [35] (Supplementary Table S2). We first mapped each RNA-Seq dataset onto the Ensembl human transcriptome using Bowtie [36]. In order to accurately align each read to the human transcriptome and take into account the multi-mapping reads caused by repeats and alternative isoforms of genes shared exons, we enabled Bowtie to search multiple optimal alignments (see Materials and methods). We then employed MMSEQ [37] pipeline to quantify the expression levels of Ensembl genes and isoforms for each sample. To minimize the false positives of expression estimation, we excluded those genes and transcripts with posterior standard deviation greater than 1.5 which is the threshold beyond the transcripts with zero unique mapping hits. To gain insights into the expression features and dynamics of human protein complexes at the transcriptome cascade, we explored those complexes in different normal tissues and four human cancers based on the expression of their corresponding genes and transcripts.

### Distinct Expression Patterns of Protein Complexes between the Gene and Isoform Levels

**Figure 2:**
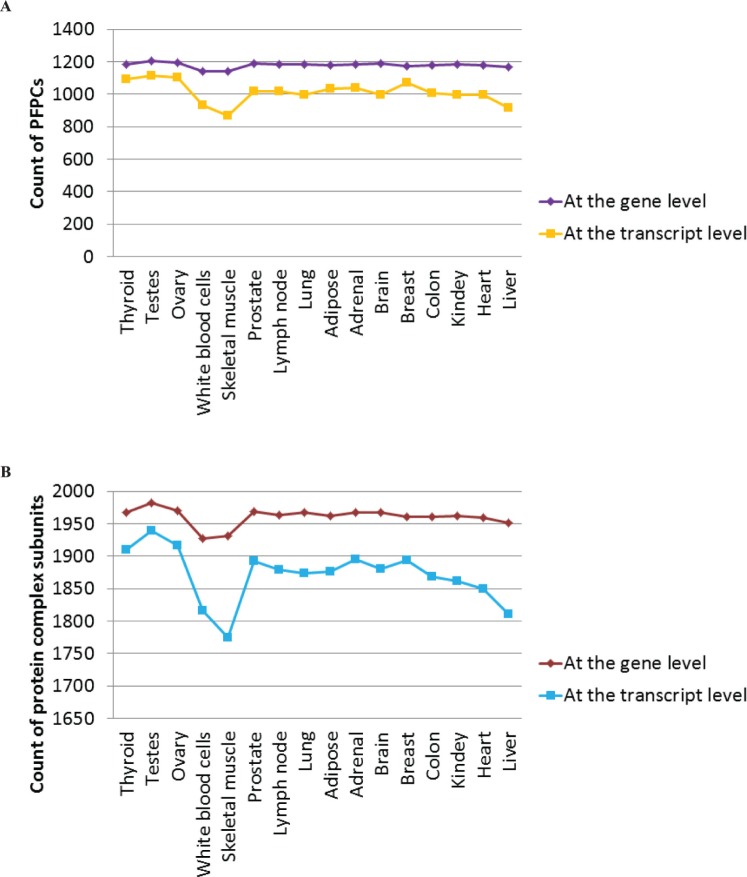
Comparison of protein complexes between the gene and transcript levels. (A) Number of PFPCs (possible formable protein complexes) at the gene and isoform levels in 16 different human tissues. (B) Count of the expressed protein complex components at the gene and transcript levels in 16 different human tissues.

To compare the differences of inquiring proteins between at the gene and isoform levels, we separately investigated the expression of those protein complexes in the 16 normal tissues using the gene and isoform expressions. If the associated transcripts of proteins expressed, these transcripts will be translated into proteins in principle. Only when all the subunits of a given protein complex expressed, can this complex have the chance to be assembled. Unless otherwise noted, we defined the possible formable protein complex (PFPC) in a certain sample as all of its complex members were detected in this sample. Using the expression of genes, average 1960 complex components and 1179 PFPCs could be detected in each sample, whereas fewer complex subunits (1871) and PFPCs (1013) were examined at the isoform level (Fig. 2A and B). This is mainly caused by the fact that in a particular condition, albeit genes have the potential to encode a number of distinct isoforms but the genes may not generate the isoforms encoded certain complex subunits. Moreover, using the gene expression level to study the expression of proteins at the transcriptome cascade may lead to the overestimation due to the expression abundance of genes is determined by the sum of their isoform expressions. Especially for those genes that generate both the transcripts encoding particular complex subunits and other isoforms, the expression abundances of those genes are certainly higher than the transcript expression levels of corresponding proteins. Consequently, it is important to accurately study proteins at the isoform-level resolution. However, a number of previous studies were used the gene expression abundance to characterize the related protein expression profile [11], [26].

### Dynamic Expression Profile of Protein Complexes in Different Human Normal Tissues

To characterize the dynamics of human protein complexes in distinct tissues, we analyzed the expression features of those protein complexes in 16 different tissues at the isoform-level resolution. We observed that many of the protein complex constituents which associated with at least two isoforms have >1 of their corresponding transcripts expressed in the same tissue. Testis had the maximum number (1113) of examined PFPCs, whereas skeletal muscle was detected the minimum number (870) of PFPCs. Each tissue was observed 83.51% of 1213 protein complexes are PFPCs on average, suggesting that the majority of these human complexes may be formed in different human tissues. Interestingly, the protein complexes that are not PFPCs mainly resulted from one of their complex members was not expressed. Moreover, 637 (52.51% of total complexes) common PFPCs were detected across these 16 disparate human tissues. The result implies that the assembly and disassembly of human protein complexes in different tissues have dynamics and each tissue may possess their specific protein complexes. We also found that the ratio of the expressed components in all complex subunits for each tissue is higher than the ratio of PFPCs in all complexes (at least larger than 5.4% and the biggest gap is 17.6%). We noted that one main reason for this is that most of those unexpressed protein complex subunits are separately involved in multiple distinct complexes. Previous studies suggested that the protein complex constituents of yeast are to a great extent co-expressed [40], [41]. The high expression ratio (on average 0.94) of those human protein complex components (calculated by the expression of their associated transcripts) also indicates this similar expression feature between human and yeast.

**Figure 3:**
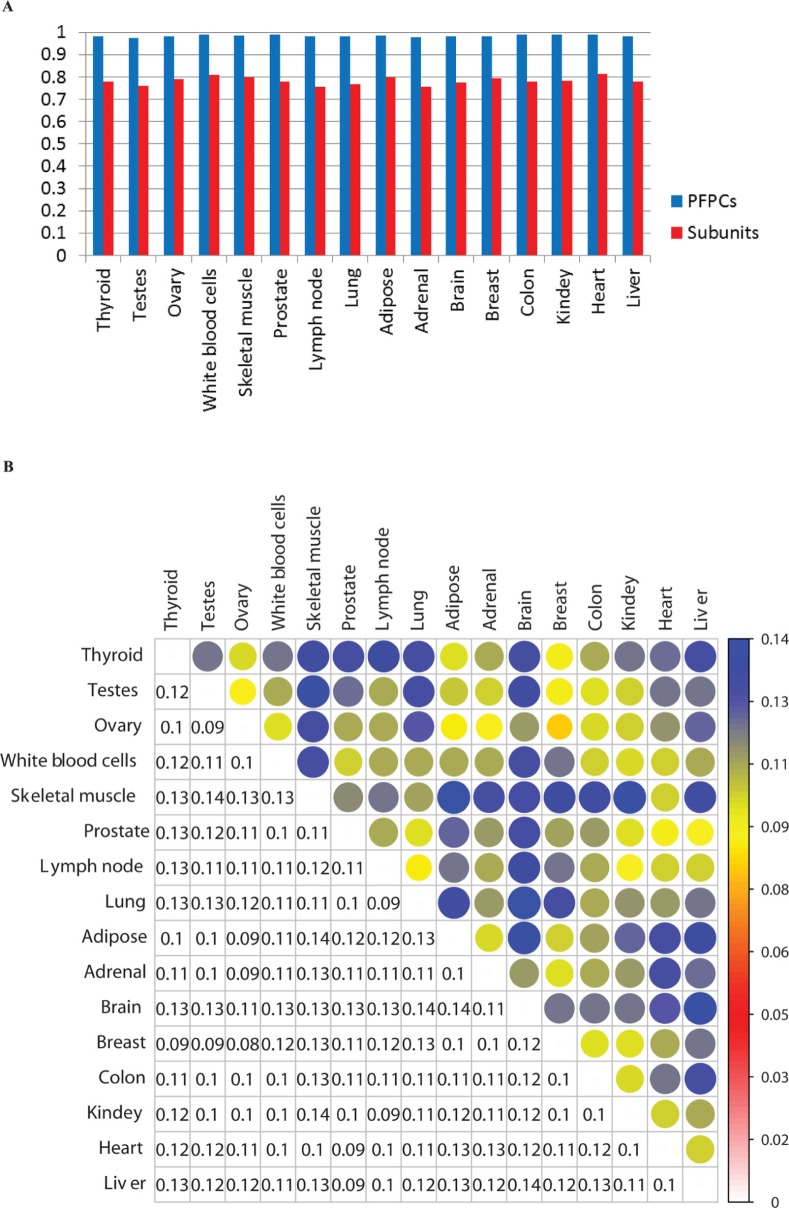
Major isoforms associated with protein complexes across different human tissues. (A) Percent for the PFPCs (blue bars) and expressed protein complex components (red bars) that involved in major isoforms in 16 human tissues. (B) Ratio of the protein complex components that switched major isoforms in the 1,123 complex constituents which possess > = 2 associated transcripts. The upper triangular matrix was shown in color method, while number method was used for the lower triangular matrix.

The vast majority of those PFPCs in different tissues contain at least one complex constituent associated with the dominant isoform of expressed gene (Fig. 3A). Greater than 97% of the PFPCs related to the major isoforms in each tissue. Moreover, average 78.25% of those expressed protein complex subunits possess the dominant isoform of genes across 16 tissues (Fig. 3A). Therefore, our finding indicates that the major isoform of the genes of complex subunits tend to be involved in the protein complex formation. We also noted switches of major isoform for those protein complex components that have at least two associated transcripts from the same gene across different tissues. Between any two of 16 disparate tissues, 8.37%–14.16% of the 1,123 complex constituents that possess > = 2 corresponding transcripts switched major isoforms (Fig. 3B). Interestingly, there is relatively lager major isoform switching rate between brain and other tissues as well as between skeletal muscle and other tissues. In total, about 35% of these complex components were found the major isoform shifting among 16 different tissues. We found that the switched isoforms of those complex constituents are generated through alternative transcription and/or alternative splicing, implying the distinct transcriptional regulation and splicing mechanism in different human tissues. Accordingly, our results reveal that the formation of human protein complexes are not only related to the presence or absence of complexes, but also associated with the switches of major isoforms for complex components.

### Intriguing Expression Profile of Protein Complexes in Human Cancers

**Figure 4:**
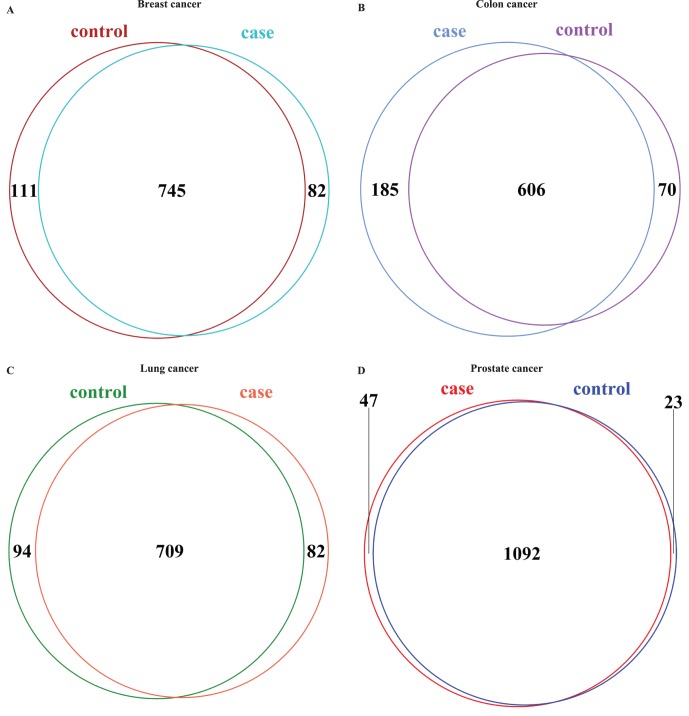
Comparison of PFPCs between cases and controls in different cancers. (A) Intersection of PFPCs between normal and cancer breast tissues. (B) Intersection of PFPCs between normal and cancer colon tissues. (C) Intersection of PFPCs between normal and cancer lung tissues. (D) Intersection of PFPCs between normal and cancer prostate tissues.

To investigate the expression changes related to protein complexes at the transcriptome cascade in human diseases, we further explored the expression variations of the associated transcripts of protein complexes between cases and controls for four human cancers of breast, colon, lung and prostate. In each type of these cancers, albeit the majority of the PFPCs between normal and disease samples are the same, a fraction of PFPCs are specific to normal or cancer tissues (Fig. 4). That is, some PFPCs in normal samples were not detected in cancer samples, while some other PFPCs were only observed in cancer. The presence and absence of the transcripts for those protein complex members would influence the assembly and disassembly of corresponding protein complexes. Owing to the crucial roles of protein complexes in human cells, a number of necessary protein complexes cannot be formed in a particular tissue may result in the tissue cells lose certain vital functions and generate disease [9], [10], [42], [43]. If a tissue constituted some unnecessary protein complexes, it also may cause abnormal changes of the tissue context. We also noted the major isoform switching for a fraction of complex components that possess > = 2 associated transcripts between cases and controls in these four human cancers.

**Figure 5:**
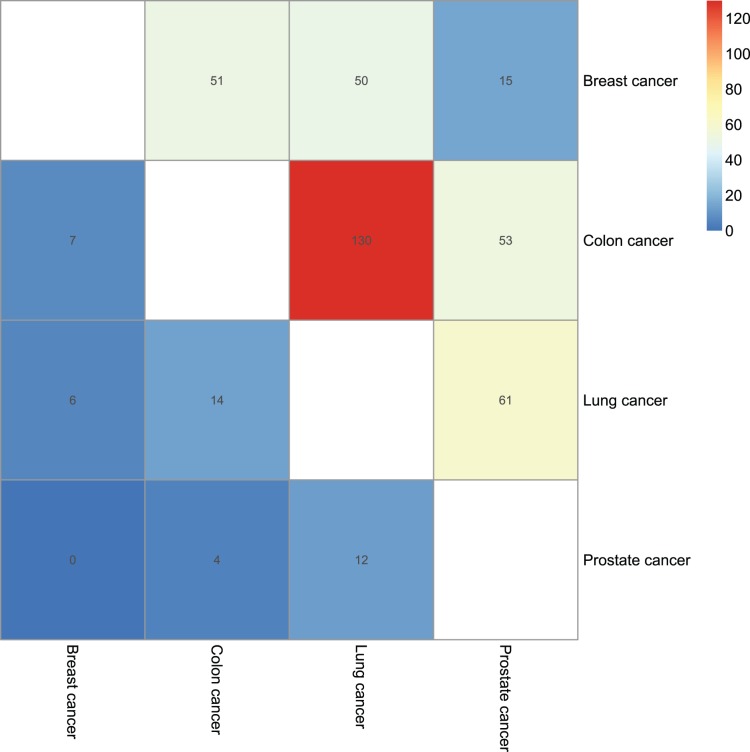
Protein complexes that related to differentially expressed transcripts in distinct cancers. The upper triangular matrix shows the shared protein complexes that associated with differentially expressed transcripts between any two of the four cancers of breast, colon, lung and prostate, whereas the lower triangular matrix shows the common differentially transcripts that are involved in protein complexes between any two of these four cancers.

We then conducted differential expression calling for these four human cancers to further interrogate the effects of differentially expressed transcripts on human protein complexes. Using NOISeq [38], we separately detected 1785, 2578, 1216 and 419 differentially expressed transcripts in the cancers of breast, colon, lung and prostate (see Materials and methods). Of these differentially expressed transcripts, 79, 152, 143 and 58 were involved in about twice protein complexes which are 178, 285, 317 and 109, respectively. It mainly result from that a portion of human proteins are shared across distinct protein complexes. Furthermore, only a few differentially expressed transcripts that involved in protein complexes were common between any two of these four cancers (the majority of them are <10), but 5–10 times more common protein complexes whose components associated with differentially expressed transcripts were observed (Fig. 5). This is caused by the fact that protein complexes usually contain multiple subunits which are related to a number of corresponding transcripts, thus any one of these associated transcripts differently expressed would affect its associated protein complex. Among these four human cancers, four common protein complexes that correlated with differentially expressed transcripts were found, but no shared differentially expressed transcripts. Taken together, these results indicate that the expression changes of transcripts may impact the expression of the proteins they encoded and further influence the formation of the protein complexes they involved. In addition, more shared properties would be observed between two disparate diseases (less shared differentially expressed transcripts but much more common affected complexes). Our findings highlights that it is important not only to study the genes and/or transcripts whose expression significantly changed, but also to investigate the protein complexes which involved by those differentially expressed transcripts.

## Discussion

Protein complexes are one of the functional module classes and they are crucial molecular entities in cells [5], [44]. We explored the expression features and dynamics of those experimentally validated human protein complexes with the RNA-Seq data from diverse human normal and cancer tissues at the transcriptome cascade. Because one gene could encode multiple distinct proteins under certain condition, it is crucial to characterize the proteins at the isoform-level resolution. This is demonstrated by our result that more protein complex constituents and PFPCs can be detected using the gene expression data than employing the expression of transcripts, which result in the overestimation at the gene level. On the other hand, a notable portion of human proteins can be encoded by several different transcripts and a small number of individual transcripts even associated with two or more Uniprot proteins (might be caused by the post-transcriptional modifications such as RNA-editing), thus the mapping relationship between proteins and transcripts is one-to-one or one-to-many or many-to-one. Of the Uniprot proteins that could be mapped to Ensembl transcripts, the proteins that possess at least two corresponding transcripts have significant advantage than those ones that only associated with single transcript in expression (the average expression ratios for these two classes of proteins among 16 human tissues are 74.61% and 38.89%, respectively). It is worth noting that a number of previous studies found higher [11]–[14] or lower [15]–[19] correlations between the gene/mRNA and protein levels, but they may fail to consider the encoded relationship between proteins and transcripts which could be one-to-many. To accurately characterize the proteins at the transcriptome cascade, it is important to map the proteins to the transcript-level resolution rather than simply to the gene-level resolution. Among those protein complexes we investigated, many of them shared one or more components, but no distinct proteins generated by the same gene are involved in distinct complexes. Using the transcript expression of related protein complex components, we observed that the human complex subunits have analogous expression property as yeast that the protein complex components are likely co-expressed in various tissues. Our results also show that the major isoforms of the genes associated with human protein complex constituents tend to be involved in complexes. On account of the complex subunits may correlate with both corresponding transcript expression and dominant isoform transition, the expression dynamics of human protein complexes not only exhibit in terms of whether the complexes are formable, but also related to the switches of major isoforms for complex members.

We found that the expression profile of human protein complexes could reveal novel insights about human diseases. In each of the four human cancers of breast, colon, lung and prostate, although the majority of PFPCs are the same between the cases and controls, a portion of PFPCs are specific to normal and cancer tissues. The absence of specific protein complexes and/or the formation of unnecessary complexes in a particular tissue may result in damages to the cells on account of the vital roles of protein complexes. In addition, because protein complexes are comprised of multiple proteins and one protein may possess several transcripts that encode this protein, therefore, albeit a few common differentially expressed transcripts that are involved in protein complexes were observed between any two of the four human cancers, much more protein complexes were found associated with differentially expressed transcripts. Each protein complex associated with a multitude of transcripts could largely increases the impacts of expression changes of their corresponding transcripts at the protein complex level. This also greatly raises the probability that two different diseases share more common properties at the protein complex level due to the expression variations of distinct transcripts would affect their common related complexes. By constructing a human disease network based on the human protein complexes, Wang et al. also found novel associations between different human diseases [9]. Therefore, investigating the expression changes regarding human protein complexes may provide us a complementary way to interpret the underlying mechanisms of human diseases.

Currently, the number of experimentally validated human protein complexes is still small and many more human protein complexes remain to be uncovered with the improvements of corresponding identification technologies. By contrast, a great portion of yeast proteins have been found involved in the assembly of protein complexes [6], [7]. The human genome is still incomplete, and the genes and proteins are also far from fully annotated [45]–[48]. Along with the progress in the annotation of human transcriptome and proteome, more genes and proteins will be identified. A portion of those unrevealed proteins may also contribute to the constituents of human protein complexes. Both the sequencing and MS technologies are undergoing fast development to improve their sensitivity and specificity, and reduce their cost. It is anticipated to simultaneously employ both RNA-Seq and MS technologies to capture the RNAs and proteins in a variety of human normal and disease tissues to comprehensively study the human protein complexes in the future.

## Supporting Information

Table S1 **The human protein complexes used in this study.**
(DOC)

Table S2 **Description of the RNA-Seq data used in this study.**
(DOC)
