# Supplementary material for: Dissecting the Characteristics and Dynamics of Human Protein Complexes at Transcriptome Cascade Using RNA-Seq Data
Source: PLoS One. 2013 Jun 18;8(6):e66521. doi: 10.1371/journal.pone.0066521 (PMC3688907; doi:10.1371/journal.pone.0066521)
Supplement: Table S2 — Description of the RNA-Seq data used in this study. (DOC) [file pone.0066521.s002.doc]

**Table S2. Description of the RNA-Seq data used in this study**

| **Tissues/Cell lines** | **Tag** | **Type of reads** | **Read length (bp)** | **Number of reads** | **Reference** |
| --- | --- | --- | --- | --- | --- |
| Thyroid | Normal | Paired-end | 50 | 81,912,887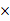2 | Illumina Human Body Map |
| Testes | Normal | Paired-end | 50 | 81,836,199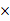2 | Illumina Human Body Map |
| Ovary | Normal | Paired-end | 50 | 80,946,260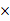2 | Illumina Human Body Map |
| White blood cells | Normal | Paired-end | 50 | 81,217,148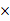2 | Illumina Human Body Map |
| Skeletal muscle | Normal | Paired-end | 50 | 82,111,139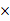2 | Illumina Human Body Map |
| Prostate | Normal | Paired-end | 50 | 82,334,076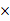2 | Illumina Human Body Map |
| Lymph node | Normal | Paired-end | 50 | 82,078,157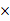2 | Illumina Human Body Map |
| Lung | Normal | Paired-end | 50 | 79,296,905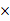2 | Illumina Human Body Map |
| Adipose | Normal | Paired-end | 50 | 77,300,072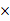2 | Illumina Human Body Map |
| Adrenal | Normal | Paired-end | 50 | 74,472,871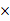2 | Illumina Human Body Map |
| Brain | Normal | Paired-end | 50 | 73,513,047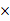2 | Illumina Human Body Map |
| Breast | Normal | Paired-end | 50 | 75,862,215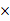2 | Illumina Human Body Map |
| Colon | Normal | Paired-end | 50 | 82,437,443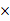2 | Illumina Human Body Map |
| Kindey | Normal | Paired-end | 50 | 80,397,337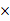2 | Illumina Human Body Map |
| Heart | Normal | Paired-end | 50 | 82,918,784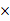2 | Illumina Human Body Map |
| Liver | Normal | Paired-end | 50 | 80,048,623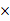2 | Illumina Human Body Map |
| Breast | Cancer  (SRR201983) | Single-end | 36 | 22,979,199 | [1] |
| Breast | Cancer  (SRR201984) | Single-end | 36 | 25,553,692 | [1] |
| Breast | Normal  (SRR201985) | Single-end | 36 | 22,864,774 | [1] |
| Breast | Normal  (SRR201986) | Single-end | 36 | 25,634,139 | [1] |
| Colon | Normal  (SRR222175) | Single-end | 65 | 9,037,384 | GSE29580 |
| Colon | Cancer  (SRR222176) | Single-end | 65 | 8,542,144 | GSE29580 |
| Colon | Normal  (SRR222177) | Single-end | 65 | 11,308,009 | GSE29580 |
| Colon | Cancer  (SRR222178) | Single-end | 65 | 11,461,875 | GSE29580 |
| Lung | Normal  (SRR192335) | Paired-end | 75 | 22,255,836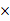2 | [2] |
| Lung | Cancer  (SRR192336) | Paired-end | 75 | 20,930,670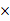2 | [2] |
| Prostate | (Normal)  ERR031017 | Paired-end | 90 | 34,536,162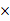2 | [3] |
| Prostate | (Cancer)  ERR031018 | Paired-end | 90 | 34,007,787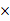2 | [3] |
| Prostate | (Normal)  ERR031019 | Paired-end | 90 | 36,250,477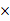2 | [3] |
| Prostate | (Cancer)  ERR031022 | Paired-end | 90 | 36,820,858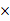2 | [3] |
| Prostate | (Normal)  ERR031023 | Paired-end | 90 | 31,245,264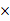2 | [3] |
| Prostate | (Cancer)  ERR031024 | Paired-end | 90 | 37,576,110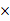2 | [3] |
| Prostate | (Normal)  ERR031025 | Paired-end | 90 | 33,918,112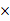2 | [3] |
| Prostate | (Cancar)  ERR031026 | Paired-end | 90 | 36,886,097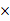2 | [3] |
| Prostate | (Normal)  ERR031029 | Paired-end | 90 | 35,534,313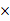2 | [3] |
| Prostate | (Cancer)  ERR031030 | Paired-end | 90 | 32,289,266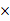2 | [3] |
| Prostate | (Normal)  ERR031031 | Paired-end | 90 | 31,921,622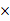2 | [3] |
| Prostate | (Cancer)  ERR031032 | Paired-end | 90 | 32,319,406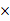2 | [3] |
| Prostate | (Normal)  ERR031033 | Paired-end | 90 | 33,965,736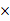2 | [3] |
| Prostate | (Cancer)  ERR031035 | Paired-end | 90 | 35,569,937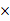2 | [3] |
| Prostate | (Cancer)  ERR031041 | Paired-end | 90 | 33,191,569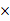2 | [3] |
| Prostate | (Cancer)  ERR031038 | Paired-end | 90 | 35,679,519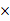2 | [3] |
| Prostate | (Normal)  ERR031039 | Paired-end | 90 | 38,401,723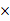2 | [3] |
| Prostate | (Cancer)  ERR031040 | Paired-end | 90 | 33,974,921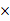2 | [3] |
| Prostate | (Cancer)  ERR031042 | Paired-end | 90 | 34,988,865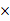2 | [3] |
| Prostate | (Normal)  ERR031043 | Paired-end | 90 | 34,266,043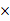2 | [3] |
| Prostate | (Cancer)  ERR031044 | Paired-end | 90 | 34,758,125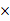2 | [3] |

**References**

1. Hon GC, Hawkins RD, Caballero OL, Lo C, Lister R, et al. (2012) Global DNA hypomethylation coupled to repressive chromatin domain formation and gene silencing in breast cancer. Genome Res 22: 246-258.
2. Beane J, Vick J, Schembri F, Anderlind C, Gower A, et al. (2011) Characterizing the impact of smoking and lung cancer on the airway transcriptome using RNA-Seq. Cancer Prev Res (Phila) 4: 803-817.
3. Ren S, Peng Z, Mao JH, Yu Y, Yin C, et al. (2012) RNA-seq analysis of prostate cancer in the Chinese population identifies recurrent gene fusions, cancer-associated long noncoding RNAs and aberrant alternative splicings. Cell Res 22: 806-821.
