# Supplementary material for: Dissecting the Characteristics and Dynamics of Human Protein Complexes at Transcriptome Cascade Using RNA-Seq Data
Source: PLoS One. 2013 Jun 18;8(6):e66521. doi: 10.1371/journal.pone.0066521 (PMC3688907; doi:10.1371/journal.pone.0066521)
Supplement: Supplementary file 2 [file pone.0066521.s001.doc]

## Sheet: human-complexes.txt.unique.Ense

Table S1. The human protein complexes used in this study
Complex ID	Complex subunits
3055	P62736,P63267,Q8TDN6,Q9NR30,Q08211,Q99848,P68104,P13639,P22087,Q9BVP2,Q92522,P16402,Q99879,P09651,P52272,Q00839,Q9NZI8,Q12905,Q12906,Q9Y383,Q9NX58,Q9BQG0,P55209,P19338,P55769,Q9Y2X3,O00567,Q14978,P06748,Q13610,Q9NW13,P27635,P62906,P62913,P30050,P26373,P40429,P50914,P61313,P18621,Q07020,Q02543,P84098,P46778,P35268,P62829,P62750,P83731,P61254,P61353,P46776,P46779,P47914,P39023,P62888,P62899,P62910,P42766,P18077,Q9Y3U8,P83881,P61513,P62891,P36578,P46777,Q02878,P18124,P62424,P62917,P32969,P05388,P05386,P05387,P62280,P25398,P62277,P62263,P62841,P62244,P62249,P08708,P62269,P15880,P62266,P62847,P62273,P61247,P62753,P62081,P62241,P46781,O76021,Q6P3W7,P05141,P37108,Q96SB4,Q13428,P11387,Q71U36,Q9H4B7,P49411,Q01081,P26368,P67809
308	P27635,P62906,P62913,P30050,P26373,P40429,P50914,P61313,P18621,Q07020,Q02543,P84098,P46778,P35268,P62829,P62750,P83731,P61254,P61353,P46776,P46779,P47914,P39023,P62888,P62899,P62910,P49207,P42766,P18077,Q9Y3U8,P83881,P61927,P61513,P63173,P62891,P36578,P62945,P46777,Q02878,P18124,P62424,P62917,P32969,P05388,P05386,P05387,P62987
193	P25786,P25787,P25788,P25789,P28066,P60900,O14818,P20618,P49721,P49720,P28070,P28074,P28072,Q99436,P62191,P35998,P17980,P43686,P62195,P62333,Q99460,O75832,O00231,O00232,Q9UNM6,O00487,Q13200,O43242,P55036,Q16401,Q15008,P51665,P48556,O00233,Q06323,Q9UL46
230	P24863,Q9BWU1,P49336,Q15648,Q9BTT4,Q9P086,Q93074,Q9UHV7,Q71F56,O60244,Q96RN5,Q9Y2X0,Q9NVC6,Q9BUE0,Q9H944,Q13503,Q15528,Q9ULK4,O75448,Q71SY5,O95402,Q6P2C8,Q9H204,Q9NX70,Q96HR3,Q9Y3C7,Q9NPJ6,O75586,O43513,Q96G25,Q9NWA0,A0JLT2
338	P62861,P46783,P62280,P25398,P62277,P62263,P62841,P62244,P62249,P08708,P62269,P39019,P15880,P60866,P63220,P62266,P62847,P62851,P62854,P62979,P62857,P62273,P23396,P61247,P62701,P46782,P62753,P62081,P62241,P46781,P08865
315	P51398,P82664,P82912,O15235,O60783,P82914,Q9Y3D3,Q9Y2R5,Q9NVS2,Q9Y676,Q9Y3D5,Q9Y399,P82921,P82650,Q9Y3D9,Q96EL2,P82663,Q9BYN8,Q92552,Q9Y2Q9,Q9NP92,Q92665,Q9Y291,P82930,P82673,P82909,P82675,P82932,Q9Y2R9,P82933
1257	O60341,Q12873,Q9P2I0,Q15029,Q13547,Q92769,O95983,Q03164,O94776,P62826,Q09028,Q15291,Q16576,O00422,O75446,Q96ST3,P51531,O60264,Q12824,Q92922,Q8TAQ2,Q92797,P21675,Q16514,P49848,Q16594,P20226,P61964
5450	Q15648,Q9BTT4,Q9P086,Q9UHV7,Q71F56,O60244,Q96RN5,Q9Y2X0,Q9NVC6,Q9BUE0,Q9H944,Q13503,Q15528,Q9ULK4,O75448,Q71SY5,O95402,Q6P2C8,Q9NX70,Q96HR3,Q9Y3C7,Q9NPJ6,O75586,O43513,Q96G25,A0JLT2,O60313
2825	P38398,P18074,P19447,Q00403,P29083,P29084,P35269,P13984,P32780,Q13888,Q13889,Q92759,Q13503,P24928,P30876,P19387,O15514,P19388,P61218,P62487,P52434,P36954,P52435,P53803,P62875,P20226
5233	O15446,O15111,Q13616,Q9UKB1,O14920,Q9Y6K9,Q13576,P52292,P42704,P23508,P46199,P19838,Q00653,Q15653,Q16342,O95602,Q9H9Y6,Q9Y2S0,Q9GZS1,P52434,P62875,Q9UJF2,Q04864,Q04206,P63208
5380	Q9UHA3,Q9UPY3,P56537,Q9HCE1,P62906,P62913,P30050,P26373,P50914,P61313,Q02543,P84098,P46778,P61353,P46779,P62888,P62910,P49207,P18077,Q9Y3U8,P61513,P18124,P62424,P05386,P05387
103	P18074,P19447,Q00403,P29083,P29084,P35269,P13984,P32780,Q13888,Q13889,Q92759,P24928,P30876,P19387,O15514,P19388,P61218,P62487,P52434,P36954,P52435,P53803,P62875,P20226
5615	P60709,Q9BZZ5,Q07021,P12277,O00148,P50402,P51858,Q02539,P61978,P14866,Q00839,Q12906,P46940,Q9Y2U8,P20700,O95777,Q13287,Q53EL6,Q08999,P62314,P10827,P62258,P27348
181	P25786,P25787,P25788,P25789,P28066,P60900,O14818,P20618,P49721,P49720,P28070,P28074,P28072,Q99436,P62191,P35998,P17980,P43686,P62195,P62333,Q9UNM6,P55036
5614	P60709,Q9H503,Q14839,Q9C005,P50402,Q13547,O15379,P68431,P20700,Q9NPJ6,Q13287,P28749,Q08999,Q9UKL0,O00422,Q12824,Q92922,Q8TAQ2,Q13813,Q9BZK7,Q9BQ87,Q13263
32	P62191,P35998,P17980,P43686,P62195,P62333,Q99460,O75832,O00231,O00232,Q9UNM6,O00487,Q13200,O43242,P55036,Q16401,Q15008,P51665,P48556,O00233
1332	Q92499,Q92841,O00571,P17844,Q8WYQ5,O43143,Q01844,P35637,P31943,P52272,Q00839,Q9BUJ2,O14979,Q12905,Q12906,Q9UKM9,Q9NRR4,Q96SB4,Q92804,Q13148
778	P60709,O96019,O14497,Q14839,Q92785,Q8WXI9,Q13547,Q92769,P07910,Q9UBB5,O95983,O94776,Q09028,P51532,Q12824,Q92922,Q8TAQ2,Q92925,Q969G3
301	P24863,P49336,Q15648,Q9BTT4,Q93074,Q9UHV7,O60244,Q9Y2X0,Q9NVC6,Q9H944,Q13503,O75448,Q6P2C8,Q9Y3C7,O75586,O43513,Q9Y2W1
470	O15265,Q92830,Q15393,O75486,O75528,Q12962,Q16514,Q15543,Q6P1X5,O00268,Q15542,O75529,P49848,Q9Y6J9,Q15545,Q16594,Q9Y4A5
642	O60341,O00257,Q9Y232,Q13363,P56545,Q9H9B1,Q96KQ7,Q13547,Q92769,Q96JN0,Q9UKL0,Q9P2K3,Q92766,P37275,O60315,O75362,Q92618
2685	Q9Y5B0,Q00403,P35269,P13984,P24928,P30876,P19387,O15514,P19388,P61218,P62487,P52434,P36954,P52435,P53803,P62875,Q9BWH6
5232	O15111,O00571,Q92896,P78347,Q9Y6K9,P41279,P19838,Q00653,P25963,Q15653,Q04864,Q04206,Q01201,P62888,Q02878,P62277,Q8NFZ5
192	P25786,P25787,P25788,P25789,P28066,P60900,O14818,P20618,P49721,P49720,P28070,P28074,P28072,Q99436,Q06323,Q9UL46
282	Q9NRL2,Q12873,Q13547,Q92769,Q9UBB5,O95983,Q13330,O94776,O60216,Q09028,Q16576,O60264,Q14683,Q9UQE7,Q8WVM7,Q8N3U4
493	P52655,P52657,Q00403,P21675,Q12962,Q15544,Q16514,Q15543,Q6P1X5,Q5VWG9,O00268,Q15542,P49848,Q15545,Q16594,P20226
529	O96019,Q9H0E9,Q9HAF1,Q9NPF5,Q96L91,Q9H2F5,Q92993,Q9NXR8,Q9UBU8,Q15014,Q9NV56,Q9Y265,Q9Y230,Q9Y4A5,Q15906,O95619
535	P24863,Q15648,Q9BTT4,Q93074,Q9UHV7,O60244,Q9Y2X0,Q9NVC6,Q13503,O75448,Q6P2C8,Q9Y3C7,Q9NPJ6,O75586,P10827,Q9Y2W1
739	O96019,O14497,Q4LE39,Q13547,Q92769,Q9UK53,Q09028,Q16576,O00422,O75446,Q96ST3,P51532,Q12824,Q92922,Q8TAQ2,Q96GM5
1141	Q14444,Q92989,Q9UKF6,Q16630,Q8N684,O60231,P19447,Q92759,P49959,O43809,O94913,P26599,P23246,Q07157,Q01081,P26368
1143	Q9UHI6,P57678,Q8TEQ6,Q8WXD5,Q9H840,Q9NWZ8,O14893,Q16637,P14678,P62314,P62316,P62318,P62304,P62306,P62308,Q9Y3F4
5199	Q16543,P07900,P08238,P34932,Q13163,Q99759,Q7KZI7,Q9NUG6,Q9UHV9,Q6Q0C0,P31946,P62258,P61981,Q04917,P27348,P63104
5613	P60709,O75531,Q9P287,Q16543,P50402,Q13283,P61978,P20700,Q9Y333,O95777,Q9H0E3,P62314,P05455,P31946,P62258,O95218
194	P25786,P25787,P25788,P25789,P28066,P60900,O14818,P20618,P49721,P49720,P28070,P28074,P28072,Q99436,P61289
232	Q9UKU7,Q15648,Q93074,Q9UHV7,O60244,Q96RN5,Q9NVC6,Q9ULK4,O75448,Q71SY5,O95402,Q9NPJ6,O75586,O43513,Q96G25
288	P24863,P49336,Q15648,Q93074,Q9UHV7,O60244,Q96RN5,Q9NVC6,Q13503,Q9ULK4,O75448,Q71SY5,O95402,O75586,O43513
492	P52655,P52657,P21675,Q12962,Q15544,Q16514,Q15543,Q6P1X5,Q5VWG9,O00268,Q15542,P49848,Q15545,Q16594,P20226
714	O96019,O14497,Q13547,Q92769,O14744,Q09028,Q96ST3,P51531,Q12824,Q92922,Q8TAQ2,Q96GM5,Q92925,Q6STE5,Q969G3
798	O96019,Q9H0E9,Q9HAF1,Q9NPF5,Q96L91,Q9H2F5,Q52LR7,Q92993,Q9NXR8,Q9UBU8,Q9NV56,Q9Y265,Q9Y230,Q9Y4A5,O95619
1068	Q9BV90,Q6IEG0,Q8N8D1,Q9H875,Q16629,P14678,P62314,P62316,P62318,P62304,P62306,P62308,Q96GM8,Q16560,P67809
5611	Q9H503,Q07021,P50402,Q02539,P68431,Q00839,P02545,P20700,P49736,P33991,Q14566,Q13287,P06400,Q08999,Q9H0E3
191	P25786,P25787,P25788,P25789,P28066,P60900,O14818,P20618,P49721,P49720,P28070,P28074,P28072,Q99436
287	P24863,P49336,Q15648,Q93074,Q9UHV7,O60244,Q96RN5,Q9NVC6,Q13503,Q9ULK4,O75448,Q71SY5,O75586,O43513
549	Q15648,Q93074,Q9UHV7,O60244,Q9Y2X0,Q9NVC6,Q9ULK4,O75448,O95402,Q9NPJ6,O75586,O43513,P28702,P11473
713	O96019,O14497,Q92769,O14744,Q09028,Q96ST3,P51532,Q12824,Q92922,Q8TAQ2,Q96GM5,Q92925,Q6STE5,Q969G3
1155	Q5TA45,Q8N201,Q9NVR2,Q96CB8,Q9H0H0,Q68E01,Q96HW7,Q6P9B9,Q9UL03,Q9NVH2,Q75QN2,Q9NV88,P24928,P30876
1230	O96019,O14497,Q9UIG0,Q13111,P51531,P51532,Q12824,Q92922,Q8TAQ2,Q96GM5,Q969G3,Q9Y5B9,Q02880,P11473
5266	Q16543,O15111,P22087,P07900,P08238,O14920,Q9Y6K9,Q99558,P62888,P36578,Q02878,P62917,P62280,P62277
116	P35226,Q14781,O00257,Q9HC52,P08107,P78364,Q8IXK0,Q8NDX5,Q06587,Q99496,Q96GD3,O60264,P25490
244	P54132,O15360,Q00597,Q9HB96,Q9NPI8,O15287,Q9NW38,P40692,Q9H9A7,P27694,P15927,P35244,Q13472
302	O96019,Q9H9F9,Q9H981,Q6PI98,Q8NBZ0,Q53TQ3,Q9ULG1,Q96EZ8,Q6P4R8,Q9Y265,Q9Y230,P15923,Q9C086
476	O15265,Q92830,Q15393,O75486,O94864,Q96BN2,O75528,Q12962,Q16514,O75529,Q9Y6J9,Q16594,Q9Y4A5
626	O60341,Q13363,Q13547,Q9NP66,Q9P0W2,P08107,Q96BD5,Q96EK2,Q9UKL0,Q9P2K3,Q92766,Q9UBW7,O75362
909	P49336,Q15648,Q93074,Q9UHV7,O60244,Q96RN5,Q9Y2X0,Q9NVC6,Q9ULK4,Q71SY5,O95402,O75586,O43513
1004	P20248,P24941,P18858,P09874,P09884,P28340,Q07864,P35251,P35250,P27694,P15927,P35244,P11387
1005	P20248,P14635,P06493,P09874,P09884,P28340,Q07864,P35251,P35250,P27694,P15927,P35244,P11387
1097	Q14152,P55884,Q99613,O15371,P60228,Q9Y262,O00303,O75821,O15372,Q13347,O75822,Q9UBQ5,Q7L2H7
1179	Q8N2Z9,Q03188,Q9H3R5,Q9BS16,Q8N0S6,Q9NSP4,Q96H22,Q9BU64,Q6IPU0,Q7L2Z9,Q96BT3,Q13352,Q71F23
1223	P62158,P27797,Q9Y262,P16104,P62805,Q8N257,O43390,P11021,P06748,P11940,P05455,Q08945,Q9Y5B9
2639	O60563,P50613,P49336,P50750,Q09472,Q92585,Q15648,P46531,P24928,Q06330,Q9BT40,Q9Y5B9,Q7KZ85
2686	P38398,P24928,P30876,P19387,O15514,P19388,P61218,P62487,P52434,P36954,P52435,P53803,P62875
3066	P24863,P49336,Q92793,P19447,Q00403,P35269,Q13889,Q13503,Q92831,P24928,P51531,P51532,Q12824
104	P24928,P30876,P19387,O15514,P19388,P61218,P62487,P52434,P36954,P52435,P53803,P62875
300	Q9BTT4,O60244,Q9Y2X0,Q9NVC6,Q9H944,Q13503,O75448,Q6P2C8,Q9Y3C7,O75586,O43513,Q9Y2W1
433	Q13315,P54132,P38398,P40692,P49959,P43246,P52701,O60934,Q92878,P35251,P35250,P35249
469	P21675,Q12962,Q15544,Q16514,Q15543,Q6P1X5,O00268,Q15542,P49848,Q15545,Q16594,P20226
485	P21675,Q12962,Q15544,Q16514,Q15543,O00268,Q92750,Q15542,P49848,Q15545,Q16594,P20226
509	P21675,Q12962,Q15544,Q16514,Q15543,Q92804,O00268,Q15542,P49848,Q15545,Q16594,P20226
514	P21675,Q12962,Q15544,Q16514,Q15543,O00268,Q15542,P49848,Q15545,Q16594,Q9HBM6,P20226
806	O96019,O14497,Q92769,O14744,Q96ST3,P51531,Q12824,Q92922,Q8TAQ2,Q96GM5,Q92925,Q969G3
1153	Q5TA45,Q8N201,Q9NVR2,Q96CB8,Q9H0H0,Q68E01,Q96HW7,Q6P9B9,Q9UL03,Q9NVH2,Q75QN2,Q9NV88
1194	O75461,Q9H9B1,Q96KQ7,O75712,Q969R5,P61244,Q8IWI9,Q9BYE7,Q06587,Q99496,Q14186,Q8IY57
2851	P29374,Q9HCU9,Q5PSV4,Q13547,Q92769,Q9H160,Q09028,Q16576,Q9H0E3,O75446,Q96ST3,Q9H7L9
3075	Q9UBL3,Q86XN7,O14686,Q8NEZ4,Q86UW6,Q14686,Q6ZW49,Q15291,Q5H9R7,O15550,P61964,Q9Y2X9
5193	O15111,Q9Y6K9,O00505,P19838,Q00653,P25963,Q15653,O00221,Q04864,Q04206,Q01201,Q8NFZ5
5196	O15084,Q9Y297,O15111,Q13616,Q14164,Q00653,O00743,Q04864,Q04206,Q9UPN7,O75170,P63208
5230	O15111,P35606,Q9Y6K9,P19838,Q00653,P25963,O00221,Q04864,Q04206,O60271,Q15025,Q8NFZ5
284	Q15648,O60244,Q96RN5,Q9NVC6,Q13503,Q9ULK4,O75448,Q71SY5,O95402,O75586,O43513
441	P03372,Q92830,Q15648,Q93074,Q9UHV7,O60244,Q9Y2X0,Q9NVC6,Q9ULK4,O75448,Q9Y4A5
633	O60341,Q14687,P78347,Q13547,Q92769,Q9P0W2,Q96BD5,Q9UKL0,Q9UBW7,Q14202,O75362
789	Q9Y2L1,Q9Y3B2,Q01780,Q13868,Q9NQT5,Q9NPD3,Q9NQT4,Q5RKV6,Q15024,Q96B26,Q06265
2739	P54132,O15360,Q8NB91,Q00597,Q9HB96,Q9NPI8,O15287,Q9NW38,Q9H9A7,P27694,Q13472
2859	O95696,P55201,Q9ULD4,Q9HAF1,Q8WYH8,O95251,Q92794,Q8WYB5,Q9NQC1,Q92613,Q6IE81
3040	P14868,O43324,P07814,P41252,Q13155,Q15046,Q9P2J5,P56192,P47897,P54136,Q12904
3082	Q92841,P17844,Q8WYQ5,Q08211,P35637,P31943,O43390,Q00839,P11021,Q12906,P19338
5177	P12814,P56945,P12830,P35222,P14923,P98161,Q05397,P49023,P12931,Q9Y490,P18206
86	P60709,O14497,Q86X55,Q96KG9,P51532,Q12824,Q92922,Q8TAQ2,Q96GM5,Q969G3
123	Q99575,O95707,Q969H6,O75817,O95059,Q9H633,Q9BUL9,P78346,P78345,O75818
304	O96019,Q9GZN1,Q96CJ1,P0C0S5,Q9Y265,Q9Y230,Q6ZRS2,Q15906,O95619,O43257
438	P38398,Q92830,P43246,P52701,O75486,O75528,Q12962,O75529,Q16594,Q9Y4A5
477	Q92831,O75486,O75478,O75528,Q12962,Q16514,O75529,Q9Y6J9,Q16594,Q9Y4A5
799	O96019,Q9H0E9,Q9NPF5,Q96L91,Q9H2F5,Q9Y265,Q9Y230,Q6ZRS2,Q9Y4A5,Q15906
822	Q8IU60,Q01780,Q13868,Q9NPD3,O95453,Q92900,Q9HAU5,Q9BZI7,Q8IZH2,Q9H0D6
867	Q15648,Q9P086,Q9NVC6,Q9BUE0,Q15528,Q71SY5,Q9NX70,Q9NPJ6,Q96G25,A0JLT2
910	O60244,Q96RN5,Q9Y2X0,Q9NVC6,Q9BUE0,Q9ULK4,O75448,Q9NPJ6,O75586,O43513
1029	P51946,P50613,P18074,P19447,P32780,Q13888,Q13889,Q92759,Q6ZYL4,P51948
1149	Q9Y294,Q9NVP2,Q13111,Q13112,O14929,P68431,P62805,Q8TEX9,P49321,Q09028
1227	P27797,P16989,P16104,P33778,Q99879,P62805,Q8N257,P11021,P06748,Q96EY7
1239	O96019,Q8NFD5,Q03111,P51532,Q12824,Q92922,Q8TAQ2,Q96GM5,Q92925,Q969G3
1252	O96019,O14497,Q03111,P51532,Q12824,Q92922,Q8TAQ2,Q96GM5,Q92925,Q969G3
1413	O15379,O75376,Q15459,Q15393,P51532,Q12824,Q92922,Q8TAQ2,Q6ZRS2,Q13263
1749	Q9UHI6,Q08211,P57678,Q00403,P24928,O14893,P14678,P08579,P63162,P20226
2183	Q92828,Q13227,O15379,O75164,P52732,O75376,O60907,Q9BZK7,Q9UPN9,Q86T24
2829	Q8NFD5,Q92793,Q9Y6Q9,Q15796,P84022,Q13485,P51532,Q92922,Q8TAQ2,Q9UPN9
5194	O15111,Q969H0,O14920,Q9Y6K9,Q99558,Q00653,Q04864,Q04206,O15027,O75604
5286	Q676U5,Q8IX12,Q16543,O15111,P07900,P08238,Q9Y6K9,Q15653,Q9UHD2,P40222
87	P57740,Q8WUM0,Q12769,Q8NFH4,Q8NFH3,Q9BW27,P52948,P55735,Q96EE3
96	Q9H1A4,Q9UM13,Q9UJX6,Q9UJX5,Q9UJX4,Q9UJX3,Q13042,Q9UJX2,P30260
144	Q10567,O43747,O75843,Q9BXS5,Q9Y6Q5,P61966,P56377,Q96PC3,Q63HQ0
646	O60341,Q12873,Q13547,Q9UBB5,O95983,O94776,Q09028,Q16576,Q9UKL0
685	Q14839,Q8WXI9,Q13547,Q92769,Q9UBB5,O95983,O94776,Q09028,Q16576
738	Q4LE39,Q13547,Q92769,Q9UK53,Q09028,Q16576,O00422,O75446,Q96ST3
781	Q13616,Q9UHV9,P19388,O94763,Q9Y265,Q9Y230,P63208,Q13309,Q9ULZ2
1003	P20248,P24941,P09884,Q14181,P35251,P35250,P40938,P35249,P40937
1114	A6H8Y1,Q92994,Q92664,Q12789,Q8WUA4,Q9Y5Q9,Q9UKN8,Q9Y5Q8,P20226
1150	Q9Y294,Q9NVP2,P84243,O14929,P54198,P62805,Q8TEX9,P49321,Q09028
1237	O96019,O14497,P51531,P51532,Q12824,Q92922,Q8TAQ2,Q96GM5,Q969G3
5609	P60709,O75531,P50402,Q13547,O15379,Q02539,P68431,Q8WWI1,P28749
36	Q10567,O43747,O75843,Q9BXS5,Q9Y6Q5,P61966,P56377,Q96PC3
162	Q8WTW3,Q14746,Q96JB2,Q9H9E3,Q9UP83,Q9Y2V7,P83436,Q96MW5
274	O60921,O60671,O75943,Q99638,P35250,P40938,P35249,P40937
443	O14867,P19447,O95452,Q13118,P50539,P17980,O15198,Q15582
696	Q9HCU9,Q13547,Q92769,Q09028,Q16576,O75446,Q96ST3,O75182
770	Q13838,Q96FV9,Q8NI27,Q96J01,Q86V81,Q13769,Q86W42,Q6I9Y2
785	Q13838,P38919,P61326,Q9Y5S9,Q15287,Q8IYB3,Q86V81,Q9BZI7
787	O96019,Q9NPF5,Q9H2F5,Q92993,Q9UBU8,Q9Y265,Q9Y230,Q9Y4A5
835	P54105,P14678,P62314,P62316,P62318,P62304,P62306,P62308
848	Q4VCS5,Q8IY63,Q9Y2J4,Q68EM7,Q8NI35,O75970,Q8N3R9,Q5T2T1
1178	Q6W2J9,Q8NHM5,P11142,Q9BSM1,Q06587,Q99496,Q8N488,P63208
1746	P57678,P09382,P17931,O14893,Q16637,P62314,P62316,P62318
2600	O60885,O60563,P50750,Q15648,Q93074,O60244,Q9NVC6,O75448
2715	Q99627,Q13616,Q13617,Q13618,Q13098,P62877,P63208,Q13309
2815	O14867,Q99728,P38398,P49959,O60934,Q92878,Q99708,Q92547
3015	P24864,P24941,P46527,P61024,Q13616,P62877,P63208,Q13309
3067	P24863,P51946,P49336,P35269,Q13503,Q12824,Q92922,Q8TAQ2
3070	Q8WVB6,P0CG12,Q9BVC3,Q9Y253,P35250,P40938,P35249,P40937
5183	P05198,P20042,P41091,Q12905,Q12906,P78527,P13010,P12956
5212	Q9H6S1,Q16543,P07900,P08238,Q92844,Q9UHD2,A7MCY6,Q12933
5268	Q16543,Q13451,P07900,P08238,O43318,Q15750,Q9NYJ8,Q8N5C8
5604	P60709,Q15417,P50402,Q8WWI1,P35579,Q12965,Q9BRV8,Q13813
27	P61160,P61158,O15143,O15144,O15145,P59998,O15511
49	Q9UBC3,Q13547,O95239,Q96ST3,O60264,O95347,Q9NTJ3
59	O00203,Q13367,O14617,Q9Y2T2,P53677,Q92572,P59780
61	Q14839,Q13547,Q92769,O95983,O94776,Q09028,Q16576
159	Q15021,Q9BPX3,Q15003,P09874,O95347,Q9NTJ3,P18887
229	P24863,P49336,Q9BTT4,O60244,Q13503,Q9ULK4,O75586
561	O15116,Q9Y333,P62310,Q9Y4Z0,Q9Y4Y9,P62312,Q9UK45
562	Q9Y333,P62310,Q9Y4Z0,Q9Y4Y9,P62312,Q9UK45,O95777
570	O14497,Q92793,Q09472,P51532,Q12824,Q92922,Q8TAQ2
614	O60341,Q12873,Q14839,Q13547,Q92769,Q13330,Q09028
620	O60341,Q13547,Q92769,Q9P0W2,Q96BD5,Q9UKL0,O75362
732	Q13547,Q92769,Q09028,Q16576,O00422,O75446,Q96ST3
811	Q9Y5B0,P84090,P35269,P30876,O14744,Q15208,Q9BQA1
814	Q9UBU9,Q9Y5S9,Q15287,Q92900,Q9HAU5,Q9H1J1,Q9BZI7
832	P54105,Q9UHI6,P57678,O14744,O14893,Q16637,P14678
871	O60341,P51587,Q13547,Q92769,Q9P0W2,Q96BD5,Q9UKL0
924	Q9NR30,Q08211,P07910,Q6P2Q9,Q96SB4,Q08945,P11388
1106	Q12789,Q8WUA4,Q9Y5Q9,Q9UKN8,Q9Y5Q8,P53999,P11387
1139	O14810,Q8WVH0,Q7Z7G2,P60880,Q16623,Q13277,P63027
1147	Q10570,Q9P2I0,Q9UKF6,Q05048,P33240,Q12996,Q92797
1189	P49917,P49959,O60934,Q92878,Q13426,P13010,P12956
1193	P49959,P09874,Q92878,Q15554,Q9NYB0,P13010,P12956
1200	P61803,P39656,Q9H0U3,P04843,P04844,Q8TCJ2,Q13454
1204	Q96AP0,Q9NUX5,Q92878,P54274,Q15554,Q9NYB0,Q9BSI4
1226	P27797,Q7L2E3,P16104,Q8N257,P11021,P06748,P09874
1256	Q9UBL3,P51610,O00255,Q03164,Q15291,P05546,P61964
1400	Q9UBL3,O14686,Q8NEZ4,Q14686,Q15291,P68366,P07437
1505	Q13547,Q92769,O15379,O75376,Q9Y618,O75446,Q96ST3
2236	Q9Y294,Q9NVP2,Q13112,O96017,P68431,P49321,P50502
2529	P22681,P62993,O43561,Q13094,P27986,P19174,P15498
2727	O15111,Q92793,O14920,Q9Y6K9,Q15596,Q9Y6Q9,P10155
2757	Q969L4,P83369,P14678,P62318,P62304,P62306,P62308
2766	P49959,Q92878,Q15554,Q9NYB0,Q9BSI4,P13010,P12956
2858	Q9HAF1,Q9UNL4,Q8WYH8,O95251,Q9NQC1,Q92613,Q6IE81
2884	P03886,P56556,Q16795,Q9Y375,O95139,O75489,O75251
3118	O14893,Q16637,P14678,P62314,P62316,P62318,P62304
5589	Q96GY3,Q52LA3,Q6MZP7,Q5TKA1,P10244,Q09028,P28749
5596	Q16254,Q96GY3,Q52LA3,Q6MZP7,Q5TKA1,Q09028,Q08999
15	Q92828,Q13227,O15379,O75376,O60907,Q9BZK7
112	O60925,Q9UHV9,Q9NQP4,Q99471,O15212,P61758
138	Q96AP0,Q9NUX5,P54274,Q15554,Q9NYB0,Q9BSI4
310	P14635,O95067,P24385,P06493,P38936,P12004
387	P49736,P25205,P33991,P33992,Q14566,P33993
389	Q13415,Q13416,Q9UBD5,O43929,O43913,Q9Y5N6
486	P62736,P19105,P35579,P60660,P42768,O43516
636	O60341,Q13547,Q92769,Q9P0W2,Q96BD5,Q9UKL0
834	P54105,O14744,P14678,P62314,P62316,P62318
927	Q03188,Q9H3R5,Q9NSP4,Q96H22,Q96BT3,Q71F23
1113	Q92664,Q12789,Q8WUA4,Q9Y5Q9,Q9UKN8,Q9Y5Q8
1182	O75934,Q99459,Q92616,P11142,O43660,Q9UMS4
1199	P61803,P39656,Q9H0U3,P04843,P04844,P46977
1206	Q9NUX5,P54274,Q15554,Q9NYB0,Q9BSI4,O95271
1211	P35869,P27540,Q13620,Q16531,P62877,Q12788
1474	Q16254,Q15329,P28749,P84022,Q13485,Q14186
2203	O60885,P35251,P35250,P40938,P35249,P40937
2217	Q13315,Q9BXW9,Q14676,P49959,O60934,Q92878
2235	Q9Y294,Q9NVP2,Q13111,Q13112,P54198,P68431
2599	O60563,P50750,Q9P2I0,P19338,P24928,Q9UBK2
2638	P50613,Q92793,Q09472,P24928,Q06330,Q7KZ85
2657	P51946,P50613,P03372,Q92769,P51948,Q13330
2730	Q9UBL3,Q8ND76,Q15291,Q9UPS6,P61964,Q6UXN9
2731	Q9UBL3,Q8ND76,Q15291,O15047,P61964,Q6UXN9
2738	P54132,Q9H9A7,P27694,P15927,P35244,Q13472
2797	Q8WVB6,P12004,P35250,P40938,P35249,P40937
2817	O14867,Q99728,P38398,P40692,P52701,Q92547
2837	P63261,Q00610,P11142,P07737,Q9BVA1,P55072
2939	Q9BQ95,P00403,P03886,O15239,Q9Y375,Q9Y4K3
3102	P55265,Q08211,Q00341,P78527,P13010,P12956
3167	Q13547,Q92769,Q9UBX0,Q9Y618,Q96ST3,O75182
4999	Q9BUN8,Q9GZP9,Q9UBV2,Q9BQE4,Q86TM6,P55072
5197	P54132,P49959,O60934,Q6ZW49,Q92878,Q12888
5209	P25786,P25787,P60900,Q05086,Q9UMX0,Q9UHD9
5251	Q13416,Q9UBD5,O43929,Q9Y5N6,P13010,P12956
5269	Q16543,Q13451,P07900,P08238,Q14164,Q86UP2
5378	Q9UPY3,Q9UKV8,P56537,Q9HCE1,P62424,Q15633
5495	P51946,P50613,P18074,P19447,P32780,Q13888
5608	P60709,P50402,P02545,P20700,Q13287,Q13813
41	P41182,Q13547,O95983,O94776,Q9BTC8
58	Q13227,O15379,Q9Y618,O60907,Q9BZK7
114	Q16533,Q13487,Q92966,Q5SXM2,O75971
142	Q96BI3,P35613,Q92542,P49768,Q9NZ42
157	Q15021,Q9BPX3,Q15003,O95347,Q9NTJ3
160	P42695,Q86XI2,Q6IBW4,O95347,Q9NTJ3
186	Q8IZP0,Q8WUW1,Q7L576,Q9Y2A7,Q9Y6W5
207	Q96Q27,Q93034,Q9UBF6,Q15369,Q15370
208	Q9Y576,Q93034,Q9UBF6,Q15369,Q15370
209	Q9NWX5,Q93034,Q9UBF6,Q15369,Q15370
210	Q9H672,Q93034,Q9UBF6,Q15369,Q15370
211	Q8WXK4,Q93034,Q9UBF6,Q15369,Q15370
214	Q93034,P62877,Q15369,Q15370,Q9Y6I7
261	O43502,O15315,O75771,O43543,O43542
270	O75943,P35250,P40938,P35249,P40937
280	P04406,P09429,P26583,P11142,P30101
297	P12004,P28340,P49005,Q15054,Q9HCU8
313	P24385,P30281,Q00535,P38936,P12004
376	P40692,P43246,P52701,P12004,P54278
525	O96019,Q92993,Q9Y265,Q9Y230,Q9Y4A5
585	P41182,Q14839,Q13547,O95983,Q9BTC8
634	O60341,P78347,Q13547,Q92769,Q14202
657	Q13596,O60749,O75436,Q9UBQ0,Q96QK1
741	Q13227,O15379,O75376,O60907,Q9BZK7
753	Q03135,Q14118,P29474,O43556,P46939
774	Q96FV9,Q8NI27,Q13769,Q86W42,Q6I9Y2
877	O14662,O43752,O75379,Q96AJ9,Q9UEU0
938	P68400,P19784,P67870,Q08945,Q9Y5B9
974	Q6ZN18,O75530,Q15910,Q09028,Q15022
994	O43292,Q92643,Q96S52,Q969N2,Q9H490
996	O75530,Q15910,Q09028,Q16576,Q15022
1037	P19447,P32780,Q13888,Q13889,Q92759
1060	Q13596,O60749,Q4G0F5,Q9UBQ0,Q96QK1
1085	P49916,Q969S2,O00764,P06746,P18887
1086	P49916,Q96FI4,O00764,P06746,P18887
1105	Q12789,Q8WUA4,Q9Y5Q9,Q9UKN8,Q9Y5Q8
1137	O14810,Q8WVH0,P60880,Q16623,P63027
1138	Q8WVH0,Q7Z7G2,P60880,Q16623,P63027
1144	Q10570,Q9P2I0,Q9UKF6,O95639,Q6UN15
1162	Q13619,Q13620,Q16531,Q92466,P62877
1167	Q6P1J9,Q6PD62,Q8WVC0,Q8N7H5,Q9GZS3
1170	O96019,P01106,Q9Y265,Q9Y230,Q9Y4A5
1176	Q09161,P52298,P62826,Q9H814,O14980
1191	P50750,O43719,P19338,P24928,O00267
1214	Q13619,Q16531,Q7L5Y6,P62877,Q8NHY2
1261	P08621,P09661,Q8IYB3,Q9UQ35,P62995
1345	Q9NVA2,Q15019,Q16181,Q92599,Q9UHD8
1372	Q86U70,P25791,P06400,P17542,P15923
1874	Q9H115,P60880,Q86Y82,P63027,Q15836
2187	Q9Y297,Q13616,Q9UKB1,P25963,P63208
2188	Q9Y297,Q13616,Q15843,P62877,P63208
2189	Q9Y297,Q13616,P62877,P63208,P84022
2211	Q99728,P38398,Q05048,P33240,Q12996
2224	P54132,P43246,P52701,Q06609,P04637
2318	P23229,P16144,O15230,P07942,P11047
2319	P23229,P16144,O15230,P55268,P11047
2745	O95999,Q9UDY8,Q9NYJ8,Q9Y4K3,Q13404
2799	P35251,P35250,P40938,P35249,P40937
2922	P62993,O43561,P27986,P19174,Q07889
2936	Q9BQ95,P04406,P00403,Q9Y375,Q9Y4K3
2975	Q16254,Q15329,P28749,P84022,Q14186
3036	P61024,Q13616,P62877,P63208,Q13309
3045	P19838,P14859,P09086,Q04206,P25490
3071	Q8IUR7,Q7L5Y9,Q9UL63,Q96S59,Q9H871
3083	O15360,Q00597,Q9HB96,Q9NPI8,O15287
3271	Q96BI3,O00548,Q92542,P49768,Q9NZ42
3284	Q9UHI6,P57678,Q8TEQ6,O14893,Q16637
3551	Q8WTW3,Q14746,Q96JB2,Q9H9E3,Q96MW5
3552	Q8WTW3,Q9UP83,Q9Y2V7,P83436,Q96MW5
4392	Q14152,P55884,Q99613,O75821,Q13347
4403	Q14152,P55884,O75821,Q13347,O75822
5117	P26358,Q16254,Q13547,Q08999,O43463
5118	Q16254,Q09472,Q13547,Q08999,O43463
5179	Q14686,P09874,P78527,P13010,P12956
5184	Q13547,P51608,Q96ST3,P51531,Q969G3
5211	Q13526,P62714,P30153,P63151,P04049
5220	Q9ULX6,O15111,Q13576,Q04206,Q8NFZ5
5228	P41279,P51003,Q04864,Q04206,Q8NFZ5
5231	P27694,P15927,Q12888,P13010,P12956
5234	Q16543,P07900,P08238,O14920,Q8N163
5260	P35222,Q09472,Q9UBN7,P63165,P15884
5283	Q92989,Q8WW01,Q8NCE0,Q9BSV6,Q7Z6J9
5285	Q16543,O15111,Q13451,P08238,Q15653
5400	P38398,P51587,P46736,Q9NXR7,Q06609
5449	P35659,Q9Y5S9,Q15287,Q8IYB3,Q86V81
5564	P40189,P23458,P61968,Q06124,O14543
5593	Q96GY3,Q52LA3,Q6MZP7,Q5TKA1,Q09028
5877	P15056,Q8IVT5,Q02750,P04049,P62258
5876	P60842,Q9Y570,P67775,P30153,P30154
4	Q92793,Q09472,Q9Y6Q9,Q92831
45	Q96BI3,Q92542,P49768,Q9NZ42
67	Q9Y6B7,Q9UPM8,O00189,Q9Y587
81	Q14999,Q8N3Y1,P62877,P63208
91	O15360,Q00597,Q9NPI8,O15287
100	Q12830,Q09028,Q16576,P28370
118	P37287,Q92535,Q14442,Q9BRB3
127	O14777,Q9BZD4,Q8NBT2,Q9HBM1
140	Q9BXY8,Q86U70,P25791,Q02577
165	O60216,Q14683,Q9UQE7,Q8WVM7
166	O60216,Q14683,Q9UQE7,Q8N3U4
190	O60566,O43684,Q12834,Q13257
234	Q9NRL2,Q9NRG0,Q9NRF9,O60264
246	P54132,P40692,Q9H9A7,Q13472
251	O43502,O15315,O75771,O43543
311	P24385,P24941,P38936,P12004
312	P24385,P11802,P38936,P12004
362	P49916,Q96T60,P06746,P18887
370	P40692,P43246,P52701,P54277
467	Q8WW43,Q92542,P49810,Q9NZ42
468	Q96BI3,Q92542,P49810,Q9NZ42
522	P12830,P35222,Q14254,P98161
557	Q03135,P29992,Q14573,P48995
605	Q99595,O60830,O14925,O43615
610	Q7Z3B3,Q9HCI7,Q8N5Y2,Q9H7Z6
613	Q8WVB6,P0CG12,Q9BVC3,P40938
622	Q13617,Q15369,Q15370,P40337
627	P49959,O60934,Q92878,Q9Y4A5
639	Q9NWU2,Q9UL63,Q99549,Q96S59
678	Q14691,Q9Y248,Q9BRX5,Q9BRT9
729	Q13616,Q5XUX0,P62877,P63208
745	Q92769,Q9Y618,Q96ST3,O75182
749	Q13547,Q92769,P51608,Q96ST3
793	O14810,P60880,Q16623,P63027
794	Q6PUV4,P60880,Q16623,P63027
819	P54105,O14744,P62314,P62318
874	O43752,Q15836,O75379,Q9BV40
964	P38398,P49959,O60934,Q92878
1030	P51946,P50613,P18074,P51948
1051	Q13616,P62877,P63208,Q13309
1052	Q13616,Q9UKB1,P62877,P63208
1054	P20749,P03372,Q9Y6Q9,Q04206
1087	Q96GD4,O15392,O60447,Q9NQS7
1091	P48357,Q13596,O60749,O95219
1093	P06213,Q13596,O60749,O95219
1095	P00533,Q13596,O60749,O95219
1096	P16234,Q13596,O60749,O95219
1104	Q13596,O60749,O95219,P02786
1119	Q96GD4,O15392,Q53HL2,Q9NQS7
1148	P17844,Q15233,P23246,P09012
1169	O00161,Q12846,Q15836,Q9BV40
1185	P00533,P04626,O00443,O00750
1198	Q96AP0,Q9NUX5,Q15554,Q9BSI4
1201	Q96AP0,Q9NUX5,P54274,Q9BSI4
1253	Q13616,Q969H0,P62877,P63208
1255	Q9HB71,Q8IUQ4,P63208,O60907
1258	Q8N3Y1,Q92990,P62877,P63208
1260	Q13616,Q86XK2,P62877,P63208
1352	Q9HAF1,Q9UNL4,O95251,Q6IE81
1377	Q8N9N2,Q9H1I8,Q8N3C0,Q15650
1379	P10619,P34059,P16278,Q99519
1380	Q6IA86,Q9H9T3,Q96EB1,O95163
1571	Q00341,P78527,P13010,P12956
1750	Q9UHI6,P57678,P60510,Q9NY27
1826	Q9UM13,P12830,Q14511,P84022
1827	P29590,Q15796,P84022,O95405
2172	Q9H211,Q13619,Q16531,P62877
2191	Q13616,Q8NFZ0,P62877,P63208
2192	Q13616,P62877,P63208,Q86WB0
2196	O60921,P18858,O60671,Q99638
2197	P39748,O60921,O60671,Q99638
2198	O60921,P06746,O60671,Q99638
2214	P38398,Q86U70,P61968,Q99708
2218	Q14676,P49959,O60934,Q92878
2342	P06756,P26012,P50281,P01137
2355	Q08722,P06734,P06756,P05106
2363	P06756,P05106,Q14289,P49023
2376	P02751,P08514,P05106,P21980
2377	Q08722,P08514,P05106,P12931
2383	P02751,P08648,P05556,P21980
2470	P56945,P03372,P27986,P12931
2535	P22681,P62993,Q13094,P29353
2536	P62993,Q13094,P07948,P16885
2547	O43561,Q13094,P19174,Q07889
2563	P22681,P21802,P06241,P07948
2564	P06241,P07948,P20936,P07947
2565	P06241,P06239,P07948,P11836
2577	P06213,P35568,Q07666,P27986
2589	Q9UBK2,Q08170,Q13243,Q13247
2598	Q8WX92,P18615,Q8IXH7,Q9H3P2
2601	O60885,O60563,P50750,Q15648
2692	P01100,P05412,P84022,Q13485
2707	P55316,O43524,P84022,Q13485
2719	P68400,P19784,P67870,P09429
2728	Q92793,Q15788,Q15596,P10155
2753	O95999,Q9BXL7,O15117,Q9UDY8
2754	P53539,P17535,P84022,Q13485
2772	Q16526,Q13616,Q9UKT7,P63208
2773	Q49AN0,Q13616,Q9UKT7,P63208
2786	Q99728,P38398,Q6UWZ7,Q96RL1
2787	Q99728,P38398,Q99708,Q96RL1
2808	P27695,O60921,O60671,Q99638
2842	O15169,Q9UER7,Q9H2X6,P04637
2847	Q9HAF1,Q9UNL4,O95251,Q9NQC1
2848	Q9HAF1,Q9UNL4,O95251,Q92613
2850	P02751,P08648,P05556,P48745
2896	Q08722,P08514,P05106,Q05397
2909	P12314,O43561,P16885,P43405
2931	Q13363,Q99708,Q06330,Q96T58
2955	O43561,P06239,Q13094,P19174
2998	O15169,P35222,P49841,Q15172
3032	Q9UPY3,Q9UKV8,P08238,Q15633
3044	Q13547,O75376,Q96ST3,P12755
3096	P62993,P23229,P16144,P29353
3128	Q8WW43,Q92542,P49768,Q9NZ42
3162	P00742,P13726,P08709,P10646
3166	P25054,O15169,P35222,P49841
3207	Q02410,O14936,Q12959,O14910
3263	Q13547,Q9UBP5,Q9Y618,Q96ST3
3525	Q9UP83,Q9Y2V7,P83436,Q96MW5
3961	Q13547,Q96ST3,P12755,P84022
3979	P42345,Q9BVC4,Q9BPZ7,Q6R327
4389	Q14152,P55884,O75821,Q13347
4498	Q07021,P23511,P25208,Q13952
4869	P08588,P24588,Q12959,P13861
5772	P33151,P35222,P35968,Q07157
5101	P30281,P11802,Q00534,P38936
5176	Q14676,P78527,P13010,P12956
5727	Q15573,Q53T94,Q15572,P20226
5235	P09874,Q14191,P13010,P12956
5243	P49916,Q96T60,Q9NUW8,P18887
5267	Q15369,Q15370,Q8TEY7,P40337
5385	P07814,P04406,P40429,O60506
5513	P35226,O00257,P78364,Q06587
5531	Q13490,P19438,Q15628,Q12933
5544	P14635,P06493,P24522,P12004
5545	P14635,P06493,O75293,P12004
5546	P14635,P06493,O95257,P12004
5579	P26441,P26992,P40189,P42702
5606	P60709,P50402,Q13287,Q13813
5695	Q9UIF9,P26358,Q9UBC3,Q13547
5799	Q14790,O15519,Q13158,P25445
5859	Q92851,Q14790,Q13158,P25445
5872	P15056,Q02750,P36507,P62258
12	Q969F9,Q9UPZ3,Q86YV9
60	Q00978,P42224,P52630
95	Q9BXJ9,P13010,P12956
98	Q09472,Q00987,P04637
120	P01374,Q06643,P36941
129	P42575,P78560,Q9HB75
143	O00213,P05067,Q07954
156	Q9BUN8,P57678,Q86TM6
173	P49959,O60934,Q92878
177	O15294,Q9UPV9,O60296
219	Q86VP6,Q13616,P62877
220	Q8N726,Q7Z6Z7,P06748
221	Q86VP6,Q13617,P62877
222	Q86VP6,Q13618,P62877
223	Q86VP6,Q13619,P62877
224	Q86VP6,Q13620,P62877
227	Q9Y297,Q13616,P63208
240	P38398,Q99708,Q9GZX5
243	P14635,P06493,Q15311
285	P40692,P12004,P54277
286	P43246,P52701,P12004
335	P43243,Q15233,P23246
336	Q09666,P49917,Q13426
350	P49917,Q96T60,Q13426
353	P49917,O95347,Q9NTJ3
355	P78527,P13010,P12956
359	P49917,Q9H9Q4,Q13426
368	P07992,Q92889,P43246
390	Q13616,Q9UKT8,P63208
391	Q13619,Q16531,P62877
423	Q9UQ84,P40692,P54278
424	Q9UQ84,P40692,P12004
425	Q13227,O15457,O43196
432	P08107,P29475,Q9UNE7
460	P82279,Q8NI35,Q8N3R9
461	P82279,Q96JB8,Q8N3R9
478	Q92830,O75486,Q16594
517	P24588,Q08209,P13861
519	Q92667,Q99417,P31323
521	P12830,P35222,P98161
531	P07992,Q92889,P23025
533	Q9NV31,Q96G21,O00566
536	P48995,Q13507,Q9HCX4
541	P05019,P35858,P17936
550	Q03135,P29474,Q8IVI9
552	P17181,P48551,P01574
553	Q14643,P61586,P48995
571	O14497,Q92793,Q09472
575	P41279,P19838,Q8NFZ5
577	Q14192,Q9H2X6,P04637
615	Q13616,P63208,Q13309
617	Q02410,O14936,Q9NUP9
631	O14965,Q14008,O75410
648	O60341,Q13547,Q9UKL0
668	P07550,P24588,Q12791
682	P13569,P15311,O14745
687	P07550,P13569,O14745
705	P48556,Q6NUQ1,O43264
706	O14964,P60880,Q86Y82
707	P60880,Q86Y82,P63027
720	Q13547,Q96ST3,P17947
723	Q9UBU8,Q9Y605,P06400
730	O00161,Q12846,P23763
731	O00161,Q12846,P63027
733	Q12846,P23763,P51809
747	Q13547,Q9Y618,Q96ST3
761	Q9UKV3,Q15287,O00422
786	Q15788,P08235,Q08209
797	O00161,O75558,P63027
812	Q92900,Q9HAU5,Q9H1J1
813	Q92900,Q9HAU5,Q9BZI7
815	Q6WCQ1,O14974,P61586
825	P54105,O14744,P14678
828	Q9UQB3,Q96D31,P48995
833	P54105,P62314,P62318
845	P07288,P13521,P05154
847	Q68EM7,Q8TEW0,P41743
860	P26358,Q96KQ7,P12004
875	O14662,Q15836,O75379
876	O43752,Q15836,Q96AJ9
918	Q8N3R9,Q8TEW0,Q9NPB6
930	Q14155,Q9Y2X7,Q14160
934	Q8TD19,Q08945,Q9Y5B9
955	Q6P1M3,Q9BYG5,P41743
959	Q15334,Q9BYG5,P41743
997	O60870,P12004,P27694
999	P22626,Q86W51,Q15185
1010	P51946,P50613,P51948
1045	P62826,O95149,O14980
1062	P10415,Q9NZS9,Q14790
1070	Q13596,O60749,O95219
1092	P12004,P13010,P12956
1116	Q96GD4,O15392,O14980
1134	Q13535,Q14839,Q92769
1140	O14810,Q8WVH0,Q13277
1146	Q05048,P33240,Q12996
1160	Q09472,Q9UK53,P12004
1165	Q5VTR2,O75150,P51965
1168	Q15477,Q6PGP7,Q9GZS3
1173	O96019,Q9Y265,Q9Y230
1174	Q09161,P52298,Q9H814
1177	O75530,Q15910,Q96EB6
1187	Q96H20,Q9BRG1,Q86VN1
1192	Q99816,Q9UK41,A5D8V6
1202	P54274,Q9BSI4,O95271
1219	P54274,O95271,Q9H2K2
1220	Q15554,P13010,P12956
1221	P52655,P52657,Q15554
1224	P35226,Q13618,O43791
1225	Q13618,O75367,O43791
1232	Q9UKL0,Q13127,Q96ST3
1243	Q13618,Q9UER7,O43791
1259	Q13111,Q13112,Q09028
1297	P32121,P45985,Q99683
1298	P32121,P45985,P53779
1656	P24864,P24941,P46527
1734	Q15459,Q15428,Q12874
1743	P62304,P62306,P62308
1779	Q9NPB6,P36897,P37173
1793	Q96FE5,P08138,Q9BZR6
1810	Q9Y2X7,P13612,P49023
1812	O15169,Q155Q3,Q9Y6R4
1828	O15169,P84022,P36897
1831	Q09472,Q9Y6X2,P84022
2124	O15111,P03372,Q9Y6Q9
2153	P08962,P11215,P05107
2159	P31749,Q9UKG1,P10275
2170	P49427,Q13616,P62877
2204	Q99741,Q9UJA3,Q13416
2213	Q99728,P38398,P24928
2215	P38398,P61968,Q99708
2220	P07992,Q92889,P43351
2226	P43246,P52701,Q05513
2228	P54132,O75771,O43543
2233	Q13111,Q9UIS9,Q15047
2238	Q6VMQ6,Q9UIS9,Q15047
2255	P60709,Q01518,P23528
2320	P48509,P23229,P16144
2321	P06241,P23229,P16144
2322	P23229,P16144,O15230
2343	P06756,P18084,Q03405
2345	Q14773,P06756,P18084
2346	Q13443,P06756,P18084
2347	P06756,P18084,P10451
2348	O00622,P06756,P18084
2351	P02751,P06241,P18564
2352	P06756,P18564,P10451
2353	P06756,P18564,P10600
2356	Q08722,P06756,P05106
2358	P06756,P05106,P10451
2359	Q13444,P06756,P05106
2362	Q9Y624,P06756,P05106
2364	O75077,P06756,P05106
2365	Q01955,P06756,P05106
2366	P06756,P05106,O14495
2369	P00533,P06756,P05106
2370	P21926,P08514,P05106
2374	P06756,P05106,Q16363
2378	P08514,P05106,Q9Y490
2379	Q99828,P08514,P05106
2382	Q9Y624,P08514,P05106
2384	Q13444,P08648,P05556
2395	P48509,Q13683,P05556
2398	P35613,P26006,P05556
2399	P08962,P26006,P05556
2400	P48509,P26006,P05556
2401	P26006,P05556,P07996
2411	P48509,P23229,P05556
2417	Q9Y6C2,P13612,P05556
2419	P60033,P13612,P05556
2420	P19397,P13612,P05556
2421	P13612,P05556,P19320
2422	P13612,P05556,P57087
2423	Q08722,P13612,P05556
2426	P13612,P05556,P07996
2428	P13612,P05556,P35442
2429	Q08722,P17301,P05556
2430	O15335,P17301,P05556
2431	P12111,P17301,P05556
2434	P12111,P56199,P05556
2435	P56199,P05556,P17706
2437	O00622,P23229,P05556
2440	Q13443,Q13797,P05556
2442	Q13797,P05556,P19320
2443	Q13797,P05556,P24821
2445	Q13444,Q13797,P05556
2446	O43915,Q13797,P05556
2447	O43184,Q13797,P05556
2528	P04626,Q9Y316,P29353
2534	P22681,P62993,Q13094
2537	P16885,Q13393,P17252
2542	P22681,P00533,P62993
2574	P15391,P27986,P15498
2579	Q96GD4,O15392,Q9NQS7
2590	Q14192,Q12778,Q96EB6
2604	O60563,P50750,Q13309
2670	P03372,Q00987,P04637
2693	P01100,P05412,Q13469
2694	P11308,P01100,P05412
2695	P15036,P01100,P05412
2699	P03372,Q9Y3R0,P05412
2705	P49711,P84022,Q13485
2706	P84022,Q13485,P08047
2708	P05412,P84022,Q13485
2709	Q07954,P14780,P01033
2710	P01023,P08133,Q07954
2720	P68400,P19784,P67870
2726	P18084,Q05397,P49023
2736	Q96FE5,Q9BZR6,Q9NS68
2752	O95999,Q9BXL7,Q9UDY8
2758	Q15750,Q9NYJ8,O43318
2760	O43524,P84022,Q13485
2761	Q12778,P84022,Q13485
2762	P98177,P84022,Q13485
2763	P45973,Q9UIS9,O43463
2770	P21926,P23229,P16144
2774	P16104,Q14676,Q12888
2775	Q14676,Q14683,Q12888
2783	Q99728,P38398,P33240
2788	O14867,Q99728,P38398
2809	O60921,O60671,Q99638
2812	P30876,P19387,Q9BWH6
2814	P38398,Q13547,Q92769
2818	Q99728,P38398,P51587
2819	P38398,Q13363,Q99708
2822	Q99728,P38398,P61077
2823	Q99728,P38398,P68036
2826	P06756,P05106,P04004
2830	Q15796,P84022,Q9UPN9
2834	Q15796,P84022,Q13485
2838	P54105,O14744,Q9BQA1
2846	P06756,P05106,P07996
2849	P06756,P05106,P48745
2853	P39060,P08648,P05556
2863	O15269,O15270,Q9NUV7
2877	O60885,O60563,P50750
2878	O60563,P50750,O94992
2880	Q9Y297,Q13309,P30291
2881	Q13616,P62877,P63208
2882	P12111,P08648,P05106
2885	P06756,P05556,P10451
2897	Q92585,P46531,Q06330
2910	P12314,P07948,P16885
2911	Q9Y618,Q06330,Q13573
2918	P13010,P12956,P25490
2921	Q13363,P56545,Q96T58
2923	Q13363,Q99708,Q96T58
2930	Q99708,Q06330,Q96T58
2938	Q9BQ95,O75489,Q15388
2942	Q9BQ95,Q9Y375,O75489
2944	P06239,P46531,P27986
2945	Q13642,Q06330,Q06587
2946	P46531,Q06330,P25490
2956	P22681,O43561,P19174
2960	Q08881,Q13094,P19174
2961	Q13094,P19174,P15498
2962	P56945,P46108,Q14185
2966	P63096,P81274,Q14980
2969	P42345,Q9BVC4,Q6R327
2971	Q13797,P05556,P49767
2972	Q13797,P05556,P15692
2989	P78325,Q13797,P05556
3004	P25054,O15169,P35222
3027	P01137,P36897,P37173
3028	P10600,P36897,P37173
3038	O75593,Q15796,Q13485
3043	P12643,P12644,P36894
3054	Q92769,Q05195,Q96ST3
3059	P02452,Q9UKX5,P05556
3103	P06756,P05106,P08195
3111	Q13797,P05556,P10451
3112	P08648,P05556,P10451
3115	Q14773,P08514,P05106
3117	P06756,P18084,P04004
3120	Q16633,P14859,Q04724
3129	Q08211,Q7KZF4,P42226
3153	Q86VW2,P50148,P61586
3156	Q13547,Q9Y618,Q06330
3170	O15169,Q9UER7,P04637
3172	Q00987,P49757,P04637
3197	P12755,P12757,Q13485
3204	O75376,P12755,Q15796
3205	O75376,P12755,P84022
3206	O75376,P12755,Q13485
3213	Q9UBK5,P26718,Q9BZM6
3269	Q13547,P06400,P51532
3296	Q9UHI6,P57678,Q8TEQ6
3297	Q8WXD5,Q9H840,Q9Y3F4
3298	Q8TEQ6,O14893,Q16637
3544	Q14746,Q96JB2,Q9H9E3
3545	Q9UP83,Q9Y2V7,P83436
3618	Q08211,P16104,P12956
3634	Q15788,P08235,P63279
3677	O14964,Q13671,O75886
3678	P00533,Q13671,O75886
3711	P12643,Q6WN34,Q9GZX9
3714	O95613,Q9BSJ2,Q96CW5
3739	P12755,Q15796,Q13485
3740	P12755,P84022,Q13485
3753	Q92793,Q15796,Q13485
3754	Q92793,P84022,Q13485
3900	Q01094,Q8TAK5,Q14186
3917	P43694,Q8IZQ8,P11831
3972	P12757,P84022,Q9HAU4
3980	P42345,Q9BVC4,Q8N122
4043	P27540,Q99814,Q9Y6K9
4082	Q14191,P13010,P12956
4395	P55884,O75821,Q13347
4399	P55884,Q13347,O75822
4478	P23511,P25208,Q13952
4976	Q9BUN8,Q8NHG7,P55072
4977	Q9UKV5,Q9BUN8,P55072
4997	Q9BUN8,Q9BQE4,P55072
4998	Q9GZP9,Q9BQE4,P55072
5100	P30281,P11802,Q00534
5107	P30279,P11802,Q9UHV2
5144	P20248,Q01094,P28749
5158	P51608,P51531,Q969G3
5159	P24864,Q16254,P28749
5162	P19419,P41970,P11831
5165	O43747,P09958,Q6VY07
5171	Q13191,P00533,Q96B97
5189	Q9Y6J0,P62158,P27348
5190	P98172,P29317,Q13009
5198	Q92793,P10276,P19793
5210	Q92844,Q12933,Q13114
5213	O43933,Q7Z412,Q13608
5215	O75390,Q15750,Q9NYJ8
5222	Q02750,Q9Y2Q5,Q9UHA4
5226	Q9Y2Q5,P28482,Q9UHA4
5229	P27694,P15927,P35244
5244	O60762,O94777,Q9P2X0
5261	P35222,Q09472,P15884
5264	Q92793,P35222,P15884
5270	Q15369,Q15370,P40337
5273	Q16665,P17980,P40337
5276	Q9GZT9,Q16665,Q13438
5277	Q9H6Z9,Q16665,Q13438
5280	P11717,O60664,P51151
5282	P56945,Q05397,P12931
5284	Q92989,Q8NCE0,Q7Z6J9
5288	Q99728,P04637,P12956
5293	P15036,P51532,Q12824
5317	O43464,O95835,P98170
5318	Q14247,Q9ULH1,P49023
5331	Q00987,P04637,P25490
5342	Q14185,Q92556,P63000
5343	P46108,Q14185,Q92556
5361	Q9UJX3,Q13042,P30260
5363	P35269,Q00839,P24928
5373	Q92831,O95359,Q9Y6A5
5381	Q9UPY3,Q9UKV8,Q15633
5451	Q9BUE0,Q9H944,Q9NX70
5461	Q15788,P19838,Q04206
5464	P19838,P25963,Q04206
5465	O00221,Q04864,Q04206
5466	Q15653,Q04864,Q04206
5467	P25963,Q04864,Q04206
5475	Q8N668,P25963,Q04206
5498	Q15052,Q13418,Q9HBI1
5502	O75530,Q15910,P25490
5518	P35226,P78364,Q8IXK0
5526	P10415,P62158,Q14318
5534	Q8TEW0,Q9NPB6,Q9NQ66
5535	Q8TEW0,Q9NPB6,Q01970
5541	Q13158,Q15628,Q12933
5550	P14635,P41002,P06493
5551	P14635,P06493,Q13635
5559	P20248,P06493,P24941
5582	P40189,P15018,P42702
5607	P60709,P50402,Q13287
5622	P38936,Q9UIM3,P07900
5641	P78352,P06241,Q12879
5646	O94887,O14786,Q9UIW2
5647	O94887,O14786,O75051
5648	O94887,O14786,P51805
5649	O94887,O14786,Q9HCM2
5655	O43707,Q9UQM7,Q96NW7
5656	Q15744,Q01094,P06400
5685	P60468,Q92890,P55072
5689	O14786,Q9UIW2,Q8NFY4
5696	P35968,O14786,P15692
5724	P22681,Q99963,Q96B97
5735	P84022,P36897,P37173
5736	P60709,P24928,P20226
5746	O14786,Q9UIW2,Q14563
5749	Q07817,Q14790,O15519
5755	Q9UBE0,P63165,Q9UBT2
5762	Q14194,Q8TDZ2,Q9UIW2
5798	Q14790,O15519,Q13158
5800	Q14790,Q13158,P25445
5805	Q14145,Q16236,Q96HS1
5809	P14867,P47870,P18507
5816	O14727,P55211,P99999
5832	Q9BXM7,Q8IXI1,Q9UPV9
5837	O60260,Q99497,Q9BXM7
5844	O15111,O14920,Q9Y6K9
5849	Q16543,P08238,Q5S007
5861	Q92851,Q13158,P25445
5862	Q03135,P03372,P21796
5870	O00213,Q13547,Q63HK5
5873	Q02750,P04049,P62258
5928	Q969H4,P04049,P12931
1	P41182,P56524
2	P41182,Q9UQL6
3	P41182,Q8WUI4
11	Q92902,Q9NQG7
13	Q92903,Q96NY9
30	Q06323,Q9UL46
55	P56524,P27361
57	P56524,P28482
75	Q92574,P49815
92	P10747,P19338
152	P29083,P29084
153	P35269,P13984
180	P62136,Q96QC0
201	Q99728,P38398
213	P49917,P18887
236	Q9UIG0,O60264
242	P38398,Q9BX63
253	O43502,O43542
254	Q9BWT6,Q9P2W1
256	O43502,O15315
264	P49959,Q92878
265	Q13535,Q8WXE1
314	P38936,P12004
328	P13010,P12956
330	Q15233,P23246
345	P49917,Q13426
360	Q96SD1,P78527
363	P49916,P18887
374	P43246,P52701
375	P43246,P20585
379	P40692,P54278
380	P40692,P54277
386	Q5XPI4,Q9BSL1
393	P53597,Q96I99
394	Q9P2R7,P53597
407	P33402,Q02153
408	Q02108,Q02153
419	P49354,P49356
422	P56539,Q14118
426	Q16819,Q16820
428	O96007,O96033
429	Q8NEB9,Q99570
472	P13674,P07237
474	Q7Z4N8,P07237
490	P52655,P52657
527	Q13507,Q9UBN4
558	Q9BX84,Q96QT4
595	Q8N608,Q9NZV8
623	P62072,Q9Y5J7
624	O60341,Q9UKL0
629	P54132,P62380
635	O00116,O15228
641	Q96S59,O95807
644	Q07666,Q96KQ4
655	Q00613,Q03933
660	O14925,Q3ZCQ8
672	P07550,Q12791
680	P49961,P43487
684	P28360,P55771
697	Q4LE39,Q9HCU9
702	Q9BXJ9,P36406
721	P17947,P20226
724	Q9UBU8,Q9H7Z6
746	P49715,Q9UBC0
748	O43318,Q9Y4K3
756	P06396,Q86TP1
757	P49841,Q86TP1
758	P15531,Q86TP1
763	P61326,Q9Y5S9
764	O75923,Q9HBI1
771	P15531,Q13131
783	Q96J02,Q93008
790	Q16531,Q92466
801	O00161,P32856
805	O00161,Q12846
820	O00161,O43752
836	P54105,O14744
839	Q5TKA1,P10244
862	P26358,Q96KQ7
873	O95721,Q16623
897	P24863,P49336
903	P07949,Q92529
904	Q13480,Q92529
906	P55265,P24941
915	O00161,Q16623
917	Q9BXF3,P28370
921	Q96T23,O60264
923	Q96T23,P28370
925	Q9NRL2,O60264
926	Q6P1M3,Q14160
928	Q14160,Q9ULK5
933	P25054,Q14160
936	Q08945,Q9Y5B9
947	O00311,Q9UBU7
1000	O14656,O14657
1028	P41235,P53999
1039	Q15004,P12004
1040	Q9UK53,Q15004
1042	Q15788,Q9HD15
1055	P29590,Q9UBW7
1056	P63165,Q9UBW7
1057	Q15834,Q96EZ8
1058	Q13596,Q9UNH7
1063	Q7Z6J2,O43739
1064	P80217,Q13287
1067	P01732,P06239
1069	Q9BZZ5,P09038
1080	O60583,P50750
1082	O60563,P50750
1083	O75909,P50750
1089	O14910,Q9NZW5
1117	O15392,O14980
1121	O95747,Q9H4A3
1123	Q9UEW8,Q9H4A3
1130	Q14247,O75146
1131	P30153,P35250
1132	P30153,P35249
1133	Q13535,Q92769
1159	Q13547,Q9UK53
1172	Q09161,P52298
1175	O96019,Q9Y4A5
1190	Q09161,Q96P71
1195	Q6P2Q9,Q8IYB3
1205	Q15554,Q9NYB0
1217	Q15554,Q14191
1218	P54132,Q15554
1233	Q92769,Q9UKL0
1245	P60510,Q9NY27
1248	O14727,P99999
1250	Q01094,P06400
1299	Q16584,Q7Z6J0
1300	Q16602,O60894
1338	O43524,Q92831
2053	O95999,Q9UDY8
2171	O14757,Q13619
2223	P54132,Q13472
2237	Q5U623,P08047
2256	Q7Z5R6,P62834
2323	P23229,P16144
2350	P06756,P18084
2354	P06756,P18564
2375	P02751,P21980
2381	P08514,P05106
2385	P08648,P05556
2406	P26006,P05556
... (truncated at 1000 rows)
